# Ba_5_(IO_6_)_2_: crystal structure evolution from room temperature to 80 K

**DOI:** 10.1107/S2056989021004990

**Published:** 2021-05-14

**Authors:** David Wenhua Bi, Priya Ranjan Baral, Arnaud Magrez

**Affiliations:** a Crystal Growth Facility, Institute of Physics, École Polytechnique Fédérale de Lausanne (EPFL), Switzerland

**Keywords:** Single-crystal structure, low-temperature X-ray diffraction, space-group determination

## Abstract

The crystal structure of Ba_5_(IO_6_)_2_, penta­barium bis­(orthoperiodate), has been re-investigated at room temperature based on single-crystal X-ray diffraction data. In comparison with a previous crystal structure determination by the Rietveld method, an improved precision of the structural parameters was achieved. Additionally, low-temperature measurements allowed the crystal structure evolution to be studied down to 80 K. No evidence of structural transition was found even at the lowest temperature. Upon cooling, the lattice contraction is more pronounced along the *b* axis. This contraction is found to be inhomogeneous along different crystallographic axes. The inter­atomic distances between different Ba atoms reduce drastically with lowering temperature, resulting in a closer packing around the IO_6_ octa­hedra, which remain largely unaffected.

## Abstract 2

The structure of Ba_5_(IO_6_)_2_ has been redetermined at two different temperatures, namely 298 and 80 K, with a high crystalline single-crystal. In comparison with previous determinations based on powder diffraction patterns, the present redetermination results are of greatly improved precision of the structural parameters. The ambiguity of the space-group assignment was eliminated with three-dimensional patterns from a single-crystal sample.

## Database survey   

No structural model was found before 2014, even though the compound had been known for a long time. Two different records with structural models from Rietveld refinement against polycrystalline diffraction patterns were found in both the ICSD (426639 & 237982) and PDF4 v2020 (04-021-7777 & 01-083-9132). However, those models have no information about the ADPs. No similar structure can be found in COD before April 2021. Obviously, the high-quality model presented herein will be very useful for research activities related to iodate materials.

## Chemical context   

Orthoperiodates are compounds based on the (IO_6_)^5−^ anion in which iodine is in the oxidation state +7. Penta­sodium orthoperiodate was the first synthesized by oxidation of sodium iodide in air in the presence of Na_2_O (Zintl & Morawietz, 1940 ▸). About 30 years later, ammonium orthoperiodates were studied for their anti­ferroelectric properties (Gränicher *et al.*, 1968 ▸). Penta­calcium orthoperiodate was foreseen as a stable nutritional complement of iodine for bovines (Moss &Miller, 1970 ▸). In the past three years, orthoperiodates have regained inter­est. They act as a stabilizing agent of Pickering solution, which is used to harvest cellulose nanocrystals (Liu *et al.*, 2018 ▸; Liu *et al.*, 2020 ▸) and chitin nanocrystals (Liu *et al.*, 2021 ▸) in high yield from microcrystalline samples. When used as ligands, orthoperiodates were found to enhance the stability of water oxidation catal­ysts (Chakraborty *et al.*, 2018 ▸).

Among alkaline earth orthoperiodates, *M*_5_(IO_6_)_2_ with *M* = Ca, Sr and Ba are the only ones to be reported in the literature since the 1960s. They are produced by a controlled oxidation of alkaline earth iodide at temperature above 723 K or by precipitation from alkaline earth hydroxide and periodic acid. Alkaline earth orthoperiodates are also observed during the course of decomposition of iodates, dimesoperiodates (Sanyal & Nag, 1977 ▸) and periodates (Balek & Julák, 1972 ▸). Ca_5_(IO_6_)_2_ and Ba_5_(IO_6_)_2_ are solids formed during the thermochemical production of hydrogen from water using a combination of iodine with Ca or Ba metals, respectively (Mizuta *et al.*, 1978 ▸).

The first crystallographic data on Ba_5_(IO_6_) were published in 2014 (Kubel *et al.*, 2014 ▸). Ba_5_(IO_6_)_2_ differs structurally from Ca_5_(IO_6_)_2_ and Sr_5_(IO_6_)_2_ (Hummel *et al.*, 2015 ▸). At room temperature, Ba_5_(IO_6_)_2_ exhibits ortho­rhom­bic symmetry while Sr and Ca orthoperiodates are isomorphic and crystallize with rhombohedral symmetry. The structure of Ba_5_(IO_6_)_2_ was refined from powder XRD data as single crystals were not available. The low-temperature dependence of the Ba_5_(IO_6_)_2_ structure, and in particular the evolution of the periodate (IO_6_)^5−^ anion, has not previously been reported.

## Structural commentary   

**Figure 1:**
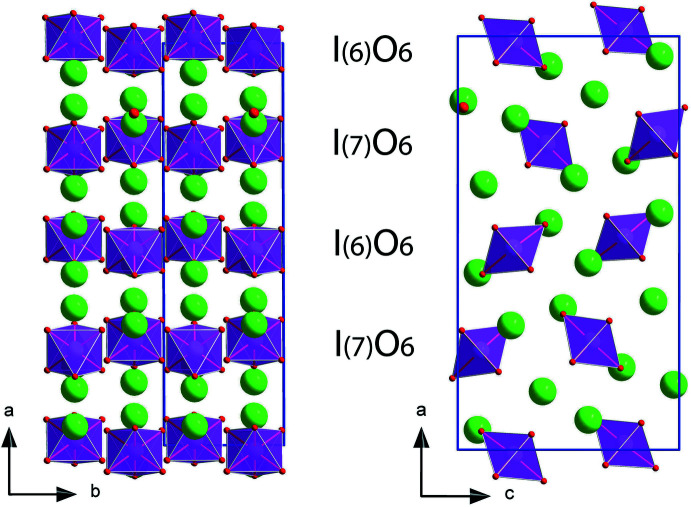
The crystal structure of Ba_5_(IO_6_)_2_ visualized along different crystallographic axes. Green, violet and red balls represents barium, iodine and oxygen atoms, respectively.

According to the comprehensive description given by Hummel *et al.*, 2015 ▸, Ba_5_(IO_6_)_2_ contains two crystallographically independent iodine atoms, which are placed in the centre of distorted octa­hedra formed by oxygen atoms, also confirmed by our room-temperature single-crystal XRD (SCXRD) data. Perpendicular to the *a* axis, the crystal structure of Ba_5_(IO_6_)_2_ is made up of alternating layers formed by I(6)O_6_ and I(7)O_6_ octa­hedra, respectively, as shown in Fig. 1 ▸. These IO_6_ octa­hedra from two consecutive layers do not share any direct connection to each other. The octa­hedra are symmetrically distributed in three dimensions over the crystal structure between the five different barium atoms of the Ba_5_(IO_6_)_2_ structure.

In order to detect any possible structural transitions in Ba_5_(IO_6_)_2_, SCXRD measurements were also performed at 100 K and 80 K. By comparing the results obtained at 80 K with those of 298 K, average thermal dilation coefficients of 4.8 × 10 ^−6^ K^−1^, 17.56 × 10 ^−6^ K^−1^, 5.23 × 10 ^−6^ K^−1^ were found along *a*, *b* and *c* axes, respectively.

**Figure 2:**
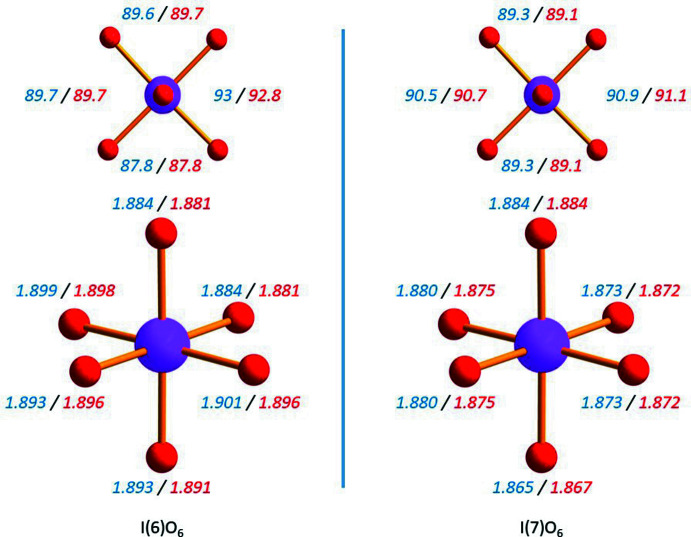
Bond lengths (in Å) and equatorial plane angles (in °) for the two IO_6_ octa­hedra of the Ba_5_(IO_6_)_2_ structure. The values obtained at 80 K and 298 K are indicated in blue and red, respectively.

**Figure 3:**
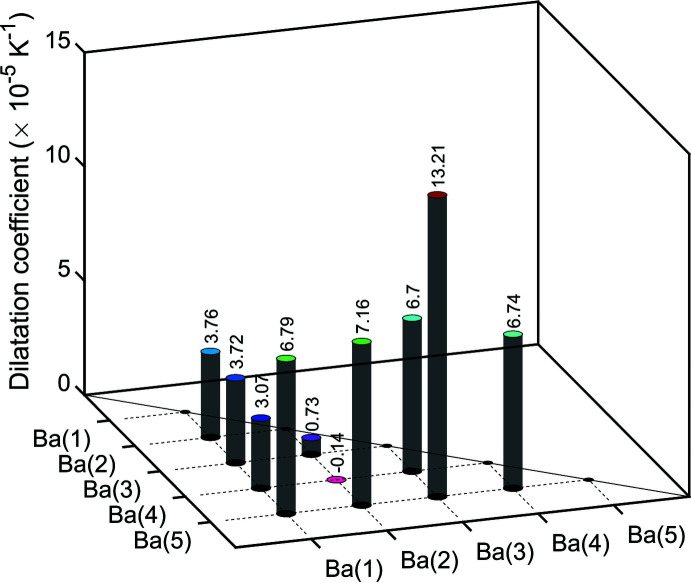
Dilation coefficient of the Ba—Ba inter­atomic distances from 80 K to 298 K.

The temperature evolution of the IO_6_ octa­hedra are quite different from the barium atom ‘matrix’ within which they are distributed. As can be seen in Fig. 2 ▸, the IO_6_ octa­hedra at 80 K are almost identical with respect to inter­atomic distances and angles to the ones refined at 298 K. However, the inter­atomic distances between Ba atoms are very sensitive to the temperature in another way. They dramatically expand when Ba_5_(IO_6_)_2_ is heated from 80 K to 298 K (Fig. 3 ▸). Except for the Ba2—Ba4 distance, all the inter­atomic distances show a dilation coefficient that is up to one order of magnitude higher than those of the lattice constant.

In conclusion, single crystals of Ba_5_(IO_6_)_2_ have been grown using a flux method. The crystal structures at two different temperatures, from 298 K down to 80 K, have been refined using high-quality SCXRD data. We confirm the assignment of the structure to the centrosymmetric space group *Pnma* (No. 62), which cannot be distinguished *via* extinction rules from the *Pna*2_1_ (No. 33) space group by powder XRD. From the low-temperature XRD data, evolution of the lattice constants was found to be inhomogeneous. While IO_6_ octa­hedra size and distortion do not change drastically between 80 K and 298 K, the packing of the barium atoms around the octa­hedra grows upon cooling. This leads to an increase of the density from 6.097 (8) g cm^−3^ at 298 K to 6.134 (7) g cm^−3^ at 80 K.

## Synthesis and crystallization   

Iron oxalate dihydrate (FeC_2_O_4_·2H_2_O), barium carbonate (BaCO_3_) and barium iodide dihydrate (BaI_2_·2H_2_O) were mixed in a molar ratio 2:2:10 and placed in an alumina crucible. The mixture was heated to 1273 K at a rate of 100 K per hour. The furnace was maintained at this temperature for 24 h, followed by a slow cooling to 1173 K at a rate of 1 K per hour, and was finally quenched to room temperature. The whole synthesis process took place under atmospheric conditions. Crystals were collected from the wall of the crucible.

## Refinement   

Crystal data, data collection and structure refinement details are summarized in Table 1 ▸.

On considering the large value of beam attenuation coefficient of the title compound, a nice crystal with small size and Mo *K*α radiation was selected for structure determination.

Data sets were collected up to the resolution of 0.6 Å at two different temperatures. No restraints were applied for the refinement at 298 K, but one O atom in the structure at 80 K had abnormal ADPs, and an EADP constraint was applied on it to eliminate the level B *checkCIF* alert. Then both models were modified to follow the same bonding and labelling style as the one in the database, and the EADP constraint was removed.

## Supplementary Material

Crystal structure: contains datablock(s) 80K, 298K. DOI: 10.1107/S2056989021004990/ru2075sup1.cif

Structure factors: contains datablock(s) 298K. DOI: 10.1107/S2056989021004990/ru2075298Ksup2.hkl

Structure factors: contains datablock(s) 80K. DOI: 10.1107/S2056989021004990/ru207580Ksup4.hkl

CCDC references: 2083048, 2083047

Additional supporting information:  crystallographic information; 3D view; checkCIF report

## Figures and Tables

**Table 1:** Experimental details

	298 K	80 K
Crystal data		
Chemical formula	Ba_5_(IO_6_)_2_	Ba_5_I_2_O_12_
*M*_r_	1132.50	1132.50
Crystal system, space group	Orthorhombic, *P* *n* *m* *a*	Orthorhombic, *P* *n* *m* *a*
Temperature (K)	298	80
*a*, *b*, *c* (Å)	19.7568 (2), 5.9003 (1), 10.5869 (1)	19.7361 (3), 5.8779 (1), 10.5749 (1)
*V* (Å^3^)	1234.13 (3)	1226.76 (3)
*Z*	4	4
Radiation type	Mo *K*α	Mo *K*α
μ (mm^−1^)	20.78	20.90
Crystal size (mm)	0.22 × 0.12 × 0.08	0.22 × 0.12 × 0.08

Data collection		
Diffractometer	XtaLAB Synergy-I with HyPix3000	XtaLAB Synergy-I with HyPix3000
Absorption correction	Gaussian (*CrysAlis PRO*; Rigaku OD, 2020 ▸)	Gaussian (*CrysAlis PRO*; Rigaku OD, 2020 ▸)
*T*_min_, *T*_max_	0.029, 0.494	0.150, 0.750
No. of measured, independent and observed [*I* > 2σ(*I*)] reflections	57653, 2543, 2407	16187, 2529, 2462
*R*_int_	0.058	0.033
(sin θ/λ)_max_ (Å^−1^)	0.769	0.769

Refinement		
*R*[*F* ^2^ > 2σ(*F* ^2^)], *wR*(*F* ^2^), *S*	0.021, 0.046, 1.16	0.022, 0.043, 1.25
No. of reflections	2543	2529
No. of parameters	104	104
Δρ_max_, Δρ_min_ (e Å^−3^)	1.39, −1.54	1.90, −1.60

Computer programs: *CrysAlis PRO* (Rigaku OD, 2020 ▸), *SHELXT2018/3* (Sheldrick, 2015*a* ▸), *SHELXL2018/3* (Sheldrick, 2015*b* ▸) and *OLEX2* (Dolomanov *et al.*, 2009 ▸).

## supplementary crystallographic information

### Pentabarium bis(orthoperiodate) (298K) Crystal data

**Table:**

Ba_5_(IO_6_)_2_	*D*_x_ = 6.095 Mg m^−^^3^
*M**_r_* = 1132.50	Mo *K*α radiation, λ = 0.71073 Å
Orthorhombic, *P**n**m**a*	Cell parameters from 29837 reflections
*a* = 19.7568 (2) Å	θ = 2.2–36.3°
*b* = 5.9003 (1) Å	µ = 20.78 mm^−^^1^
*c* = 10.5869 (1) Å	*T* = 298 K
*V* = 1234.13 (3) Å^3^	Prism, brown
*Z* = 4	0.22 × 0.12 × 0.08 mm
*F*(000) = 1928	

### Pentabarium bis(orthoperiodate) (298K) Data collection

**Table:**

XtaLAB Synergy-I with HyPix3000 diffractometer	2407 reflections with *I* > 2σ(*I*)
ω Scan scans	*R*_int_ = 0.058
Absorption correction: gaussian (CrysAlisPro; Rigaku OD, 2020)	θ_max_ = 33.1°, θ_min_ = 2.1°
*T*_min_ = 0.029, *T*_max_ = 0.494	*h* = −30→30
57653 measured reflections	*k* = −9→9
2543 independent reflections	*l* = −16→16

### Pentabarium bis(orthoperiodate) (298K) Refinement

**Table:**

Refinement on *F*^2^	0 restraints
Least-squares matrix: full	*w* = 1/[σ^2^(*F*_o_^2^) + (0.0162*P*)^2^ + 4.6175*P*] where *P* = (*F*_o_^2^ + 2*F*_c_^2^)/3
*R*[*F*^2^ > 2σ(*F*^2^)] = 0.021	(Δ/σ)_max_ = 0.001
*wR*(*F*^2^) = 0.046	Δρ_max_ = 1.39 e Å^−^^3^
*S* = 1.16	Δρ_min_ = −1.54 e Å^−^^3^
2543 reflections	Extinction correction: SHELXL2018/3 (Sheldrick 2015b), Fc^*^=kFc[1+0.001xFc^2^λ^3^/sin(2θ)]^-1/4^
104 parameters	Extinction coefficient: 0.00080 (3)

### Pentabarium bis(orthoperiodate) (298K) Special details

**Table:**

Geometry. All esds (except the esd in the dihedral angle between two l.s. planes) are estimated using the full covariance matrix. The cell esds are taken into account individually in the estimation of esds in distances, angles and torsion angles; correlations between esds in cell parameters are only used when they are defined by crystal symmetry. An approximate (isotropic) treatment of cell esds is used for estimating esds involving l.s. planes.

### Pentabarium bis(orthoperiodate) (298K) Fractional atomic coordinates and isotropic or equivalent isotropic displacement parameters (Å^2^)

**Table:**

	*x*	*y*	*z*	*U*_iso_*/*U*_eq_	
Ba1	0.04831 (2)	0.250000	0.08907 (3)	0.01014 (6)	
Ba2	0.14123 (2)	0.250000	0.38321 (3)	0.00819 (6)	
Ba3	0.19933 (2)	0.250000	0.74019 (3)	0.01149 (6)	
Ba4	0.34287 (2)	0.250000	0.47502 (3)	0.00930 (6)	
Ba5	0.42814 (2)	0.250000	0.08851 (3)	0.01175 (6)	
I6	0.01315 (2)	0.250000	0.74877 (3)	0.00578 (6)	
I7	0.23901 (2)	0.250000	0.10140 (3)	0.00659 (6)	
O8	0.03751 (13)	0.5222 (4)	0.3320 (2)	0.0132 (5)	
O9	0.18857 (14)	0.0239 (4)	0.1828 (2)	0.0141 (5)	
O10	0.28823 (15)	0.0235 (5)	0.0184 (3)	0.0184 (6)	
O11	0.43557 (13)	0.5199 (4)	0.3281 (3)	0.0134 (5)	
O12	0.07500 (19)	0.250000	0.6116 (4)	0.0135 (7)	
O13	0.3027 (2)	0.250000	0.2338 (4)	0.0182 (8)	
O14	0.45600 (19)	0.250000	0.6074 (3)	0.0123 (7)	
O15	0.6730 (2)	0.250000	0.5248 (4)	0.0178 (8)	

### Pentabarium bis(orthoperiodate) (298K) Atomic displacement parameters (Å^2^)

**Table:**

	*U*^11^	*U*^22^	*U*^33^	*U*^12^	*U*^13^	*U*^23^
Ba1	0.01190 (13)	0.00841 (12)	0.01012 (12)	0.000	−0.00023 (9)	0.000
Ba2	0.00924 (12)	0.00836 (11)	0.00698 (11)	0.000	−0.00090 (9)	0.000
Ba3	0.01612 (14)	0.00807 (12)	0.01028 (12)	0.000	0.00336 (10)	0.000
Ba4	0.01039 (12)	0.00840 (12)	0.00911 (12)	0.000	−0.00143 (9)	0.000
Ba5	0.01280 (13)	0.00815 (12)	0.01431 (13)	0.000	−0.00272 (10)	0.000
I6	0.00691 (12)	0.00474 (11)	0.00569 (11)	0.000	0.00027 (9)	0.000
I7	0.00812 (13)	0.00585 (12)	0.00581 (12)	0.000	0.00011 (9)	0.000
O8	0.0130 (12)	0.0104 (11)	0.0162 (12)	0.0040 (9)	−0.0013 (9)	0.0057 (10)
O9	0.0186 (13)	0.0092 (11)	0.0146 (12)	−0.0044 (9)	0.0066 (10)	0.0003 (9)
O10	0.0228 (14)	0.0133 (12)	0.0190 (13)	0.0074 (10)	0.0078 (11)	−0.0009 (10)
O11	0.0132 (12)	0.0086 (11)	0.0183 (12)	−0.0032 (9)	0.0038 (10)	0.0040 (10)
O12	0.0135 (17)	0.0151 (17)	0.0120 (16)	0.000	0.0082 (13)	0.000
O13	0.0192 (19)	0.0201 (19)	0.0154 (18)	0.000	−0.0103 (15)	0.000
O14	0.0114 (16)	0.0143 (16)	0.0113 (16)	0.000	−0.0035 (13)	0.000
O15	0.0130 (17)	0.029 (2)	0.0113 (17)	0.000	0.0048 (14)	0.000

### Pentabarium bis(orthoperiodate) (298K) Geometric parameters (Å, º)

**Table:**

Ba1—O8^i^	3.040 (3)	Ba4—I7^viii^	3.6209 (2)
Ba1—O8	3.040 (3)	Ba4—O9^ix^	2.799 (3)
Ba1—O9	3.232 (3)	Ba4—O9^viii^	2.799 (3)
Ba1—O9^i^	3.232 (3)	Ba4—O10^ix^	3.086 (3)
Ba1—O11^ii^	3.095 (3)	Ba4—O10^viii^	3.086 (3)
Ba1—O11^iii^	3.095 (3)	Ba4—O11^i^	2.883 (2)
Ba1—O11^iv^	2.875 (3)	Ba4—O11	2.883 (2)
Ba1—O11^v^	2.875 (3)	Ba4—O13	2.675 (4)
Ba1—O14^ii^	2.9578 (3)	Ba4—O14	2.638 (4)
Ba1—O14^vi^	2.9578 (3)	Ba4—O15^xii^	2.9668 (4)
Ba1—O14^iv^	2.766 (4)	Ba4—O15^xiii^	2.9668 (4)
Ba1—O15^iv^	2.743 (4)	Ba5—I6^ii^	3.5954 (2)
Ba2—Ba3	3.9498 (4)	Ba5—O8^ii^	3.105 (3)
Ba2—Ba5^vii^	3.9123 (3)	Ba5—O8^xiv^	2.821 (3)
Ba2—Ba5^viii^	3.9123 (3)	Ba5—O8^xv^	2.821 (3)
Ba2—I7	3.5543 (4)	Ba5—O8^iii^	3.105 (3)
Ba2—O8	2.659 (2)	Ba5—O10^i^	3.159 (3)
Ba2—O8^i^	2.659 (2)	Ba5—O10	3.159 (3)
Ba2—O9^i^	2.675 (3)	Ba5—O11	2.999 (3)
Ba2—O9	2.675 (3)	Ba5—O11^i^	2.999 (3)
Ba2—O10^viii^	2.568 (3)	Ba5—O12^vi^	2.9609 (3)
Ba2—O10^ix^	2.568 (3)	Ba5—O12^ii^	2.9609 (3)
Ba2—O12	2.749 (4)	Ba5—O13	2.917 (4)
Ba3—I7^viii^	3.5138 (2)	I6—O8^xvi^	1.881 (2)
Ba3—I7^vii^	3.5138 (2)	I6—O8^xvii^	1.881 (2)
Ba3—O9^ix^	2.808 (3)	I6—O11^vii^	1.891 (2)
Ba3—O9^viii^	2.808 (3)	I6—O11^x^	1.891 (2)
Ba3—O10^ix^	2.860 (3)	I6—O12	1.898 (3)
Ba3—O10^viii^	2.860 (3)	I6—O14^xi^	1.896 (4)
Ba3—O11^vii^	3.132 (3)	I7—O9^i^	1.875 (2)
Ba3—O11^x^	3.132 (3)	I7—O9	1.875 (2)
Ba3—O12	2.809 (4)	I7—O10^i^	1.872 (3)
Ba3—O13^vii^	2.9512 (1)	I7—O10	1.872 (3)
Ba3—O13^viii^	2.9512 (1)	I7—O13	1.883 (4)
Ba3—O15^xi^	2.542 (4)	I7—O15^iv^	1.866 (4)
Ba4—I7^vii^	3.6209 (2)		
			
O8—Ba1—O8^i^	63.79 (9)	O8^iii^—Ba5—O10	78.61 (7)
O8—Ba1—O9^i^	65.31 (7)	O8^xv^—Ba5—O10	119.97 (7)
O8^i^—Ba1—O9^i^	91.06 (7)	O8^ii^—Ba5—O10	99.72 (7)
O8—Ba1—O9	91.06 (7)	O8^xiv^—Ba5—O10	168.92 (7)
O8^i^—Ba1—O9	65.31 (7)	O8^iii^—Ba5—O10^i^	99.72 (7)
O8—Ba1—O11^iii^	173.91 (7)	O8^ii^—Ba5—O10^i^	78.61 (7)
O8^i^—Ba1—O11^iii^	122.06 (6)	O8^xv^—Ba5—O10^i^	168.92 (7)
O8—Ba1—O11^ii^	122.06 (6)	O8^xiv^—Ba5—O10^i^	119.97 (7)
O8^i^—Ba1—O11^ii^	173.91 (7)	O8^xiv^—Ba5—O11	53.68 (7)
O9^i^—Ba1—O9	48.76 (9)	O8^xv^—Ba5—O11^i^	53.68 (7)
O11^v^—Ba1—O8	88.87 (7)	O8^xv^—Ba5—O11	90.72 (7)
O11^iv^—Ba1—O8	52.77 (7)	O8^xiv^—Ba5—O11^i^	90.72 (7)
O11^v^—Ba1—O8^i^	52.77 (7)	O8^xiv^—Ba5—O12^ii^	54.84 (9)
O11^iv^—Ba1—O8^i^	88.87 (7)	O8^xv^—Ba5—O12^vi^	54.84 (9)
O11^v^—Ba1—O9^i^	143.07 (7)	O8^xv^—Ba5—O12^ii^	123.97 (9)
O11^v^—Ba1—O9	110.00 (6)	O8^xiv^—Ba5—O12^vi^	123.97 (9)
O11^iii^—Ba1—O9	90.27 (6)	O8^xv^—Ba5—O13	119.58 (8)
O11^iv^—Ba1—O9^i^	110.00 (6)	O8^xiv^—Ba5—O13	119.58 (8)
O11^iv^—Ba1—O9	143.07 (7)	O10^i^—Ba5—I6^ii^	92.63 (5)
O11^iii^—Ba1—O9^i^	111.53 (7)	O10—Ba5—I6^ii^	137.76 (5)
O11^ii^—Ba1—O9	111.53 (7)	O10^i^—Ba5—O10	50.07 (10)
O11^ii^—Ba1—O9^i^	90.27 (6)	O11^i^—Ba5—I6^ii^	91.20 (5)
O11^iii^—Ba1—O11^ii^	52.03 (9)	O11—Ba5—I6^ii^	31.71 (5)
O11^v^—Ba1—O11^iv^	67.28 (10)	O11—Ba5—O8^ii^	119.95 (6)
O11^v^—Ba1—O11^ii^	126.51 (4)	O11^i^—Ba5—O8^ii^	163.54 (7)
O11^v^—Ba1—O11^iii^	96.26 (7)	O11^i^—Ba5—O8^iii^	119.94 (6)
O11^iv^—Ba1—O11^iii^	126.51 (4)	O11—Ba5—O8^iii^	163.54 (7)
O11^iv^—Ba1—O11^ii^	96.26 (7)	O11^i^—Ba5—O10	90.96 (7)
O11^iv^—Ba1—O14^ii^	53.50 (8)	O11—Ba5—O10	117.80 (7)
O11^v^—Ba1—O14^ii^	120.68 (9)	O11^i^—Ba5—O10^i^	117.80 (7)
O11^v^—Ba1—O14^vi^	53.50 (8)	O11—Ba5—O10^i^	90.96 (7)
O11^iv^—Ba1—O14^vi^	120.68 (9)	O11^i^—Ba5—O11	64.16 (9)
O14^iv^—Ba1—O8^i^	126.15 (7)	O12^ii^—Ba5—I6^ii^	31.82 (7)
O14^vi^—Ba1—O8^i^	54.23 (8)	O12^vi^—Ba5—I6^ii^	141.76 (7)
O14^ii^—Ba1—O8^i^	118.00 (8)	O12^ii^—Ba5—O8^ii^	69.23 (9)
O14^ii^—Ba1—O8	54.23 (8)	O12^vi^—Ba5—O8^ii^	120.53 (9)
O14^vi^—Ba1—O8	118.00 (8)	O12^vi^—Ba5—O8^iii^	69.23 (9)
O14^iv^—Ba1—O8	126.15 (7)	O12^ii^—Ba5—O8^iii^	120.53 (9)
O14^iv^—Ba1—O9	142.77 (7)	O12^vi^—Ba5—O10	65.13 (9)
O14^ii^—Ba1—O9^i^	65.97 (8)	O12^ii^—Ba5—O10	115.00 (9)
O14^iv^—Ba1—O9^i^	142.77 (7)	O12^ii^—Ba5—O10^i^	65.13 (8)
O14^ii^—Ba1—O9	114.60 (9)	O12^vi^—Ba5—O10^i^	115.00 (9)
O14^vi^—Ba1—O9	65.97 (8)	O12^vi^—Ba5—O11	117.42 (9)
O14^vi^—Ba1—O9^i^	114.60 (9)	O12^ii^—Ba5—O11^i^	117.42 (9)
O14^iv^—Ba1—O11^iv^	73.66 (8)	O12^vi^—Ba5—O11^i^	53.28 (9)
O14^iv^—Ba1—O11^v^	73.66 (8)	O12^ii^—Ba5—O11	53.28 (9)
O14^vi^—Ba1—O11^ii^	119.94 (8)	O12^ii^—Ba5—O12^vi^	170.25 (15)
O14^iv^—Ba1—O11^iii^	52.89 (8)	O13—Ba5—I6^ii^	91.45 (4)
O14^ii^—Ba1—O11^ii^	67.92 (8)	O13—Ba5—O8^iii^	130.30 (8)
O14^ii^—Ba1—O11^iii^	119.94 (8)	O13—Ba5—O8^ii^	130.30 (8)
O14^iv^—Ba1—O11^ii^	52.89 (8)	O13—Ba5—O10^i^	51.70 (8)
O14^vi^—Ba1—O11^iii^	67.92 (8)	O13—Ba5—O10	51.70 (8)
O14^iv^—Ba1—O14^ii^	91.74 (7)	O13—Ba5—O11	66.15 (8)
O14^iv^—Ba1—O14^vi^	91.74 (7)	O13—Ba5—O11^i^	66.15 (8)
O14^ii^—Ba1—O14^vi^	171.78 (14)	O13—Ba5—O12^ii^	86.49 (8)
O15^iv^—Ba1—O8	115.77 (8)	O13—Ba5—O12^vi^	86.49 (8)
O15^iv^—Ba1—O8^i^	115.77 (8)	Ba1^xvi^—I6—Ba1^xviii^	109.041 (11)
O15^iv^—Ba1—O9^i^	50.55 (8)	Ba1^xvi^—I6—Ba1^xix^	66.305 (7)
O15^iv^—Ba1—O9	50.55 (8)	Ba1^xviii^—I6—Ba1^xix^	66.305 (7)
O15^iv^—Ba1—O11^ii^	61.00 (8)	Ba1^xvi^—I6—Ba3	110.289 (7)
O15^iv^—Ba1—O11^v^	146.09 (5)	Ba1^xix^—I6—Ba3	80.504 (9)
O15^iv^—Ba1—O11^iv^	146.09 (5)	Ba1^xviii^—I6—Ba3	110.289 (7)
O15^iv^—Ba1—O11^iii^	61.00 (8)	Ba5^viii^—I6—Ba1^xvi^	179.188 (10)
O15^iv^—Ba1—O14^iv^	105.18 (11)	Ba5^vii^—I6—Ba1^xviii^	179.188 (10)
O15^iv^—Ba1—O14^ii^	93.13 (7)	Ba5^viii^—I6—Ba1^xviii^	70.339 (6)
O15^iv^—Ba1—O14^vi^	93.13 (7)	Ba5^viii^—I6—Ba1^xix^	113.722 (7)
Ba1—Ba2—Ba3	166.371 (11)	Ba5^vii^—I6—Ba1^xix^	113.722 (7)
Ba1—Ba2—Ba5^vii^	107.497 (7)	Ba5^vii^—I6—Ba1^xvi^	70.339 (6)
Ba1—Ba2—Ba5^viii^	107.497 (8)	Ba5^viii^—I6—Ba3	70.481 (7)
Ba5^viii^—Ba2—Ba3	64.547 (6)	Ba5^vii^—I6—Ba3	70.481 (7)
Ba5^vii^—Ba2—Ba3	64.547 (6)	Ba5^viii^—I6—Ba5^vii^	110.276 (11)
Ba5^vii^—Ba2—Ba5^viii^	97.888 (9)	O8^xvi^—I6—Ba1^xviii^	128.23 (8)
I7—Ba2—Ba1	63.445 (8)	O8^xvi^—I6—Ba1^xix^	123.16 (8)
I7—Ba2—Ba3	130.184 (10)	O8^xvii^—I6—Ba1^xviii^	57.00 (8)
I7—Ba2—Ba5^vii^	131.052 (5)	O8^xvii^—I6—Ba1^xix^	123.16 (8)
I7—Ba2—Ba5^viii^	131.052 (5)	O8^xvii^—I6—Ba1^xvi^	128.23 (8)
O8—Ba2—Ba1	55.46 (6)	O8^xvi^—I6—Ba1^xvi^	57.00 (8)
O8^i^—Ba2—Ba1	55.46 (6)	O8^xvi^—I6—Ba3	121.37 (8)
O8^i^—Ba2—Ba3	114.77 (6)	O8^xvii^—I6—Ba3	121.37 (8)
O8—Ba2—Ba3	114.77 (6)	O8^xvi^—I6—Ba5^vii^	51.02 (8)
O8—Ba2—Ba5^vii^	52.26 (6)	O8^xvii^—I6—Ba5^vii^	122.91 (8)
O8—Ba2—Ba5^viii^	107.38 (6)	O8^xvii^—I6—Ba5^viii^	51.02 (8)
O8^i^—Ba2—Ba5^viii^	52.26 (6)	O8^xvi^—I6—Ba5^viii^	122.91 (8)
O8^i^—Ba2—Ba5^vii^	107.38 (6)	O8^xvii^—I6—O8^xvi^	91.20 (16)
O8—Ba2—I7	104.34 (6)	O8^xvi^—I6—O11^vii^	88.51 (11)
O8^i^—Ba2—I7	104.34 (6)	O8^xvii^—I6—O11^vii^	179.34 (12)
O8—Ba2—O8^i^	74.30 (11)	O8^xvii^—I6—O11^x^	88.51 (11)
O8—Ba2—O9^i^	78.85 (8)	O8^xvi^—I6—O11^x^	179.34 (12)
O8^i^—Ba2—O9^i^	114.13 (8)	O8^xvii^—I6—O12	89.69 (12)
O8—Ba2—O9	114.14 (8)	O8^xvi^—I6—O12	89.69 (12)
O8^i^—Ba2—O9	78.85 (8)	O8^xvii^—I6—O14^xi^	92.76 (11)
O8—Ba2—O12	79.19 (9)	O8^xvi^—I6—O14^xi^	92.76 (11)
O8^i^—Ba2—O12	79.19 (9)	O11^vii^—I6—Ba1^xviii^	123.62 (8)
O9^i^—Ba2—Ba1	59.62 (6)	O11^x^—I6—Ba1^xviii^	52.00 (8)
O9—Ba2—Ba1	59.63 (6)	O11^x^—I6—Ba1^xvi^	123.62 (8)
O9^i^—Ba2—Ba3	131.09 (6)	O11^vii^—I6—Ba1^xix^	57.47 (8)
O9—Ba2—Ba3	131.09 (6)	O11^vii^—I6—Ba1^xvi^	52.00 (8)
O9^i^—Ba2—Ba5^viii^	159.91 (5)	O11^x^—I6—Ba1^xix^	57.47 (8)
O9—Ba2—Ba5^vii^	159.91 (5)	O11^x^—I6—Ba3	58.35 (8)
O9^i^—Ba2—Ba5^vii^	100.78 (5)	O11^vii^—I6—Ba3	58.35 (8)
O9—Ba2—Ba5^viii^	100.78 (5)	O11^vii^—I6—Ba5^vii^	56.48 (8)
O9^i^—Ba2—I7	31.15 (5)	O11^x^—I6—Ba5^vii^	128.75 (8)
O9—Ba2—I7	31.15 (5)	O11^x^—I6—Ba5^viii^	56.48 (8)
O9^i^—Ba2—O9	59.83 (11)	O11^vii^—I6—Ba5^viii^	128.75 (8)
O9^i^—Ba2—O12	149.76 (5)	O11^vii^—I6—O11^x^	91.77 (16)
O9—Ba2—O12	149.76 (5)	O11^vii^—I6—O12	89.72 (12)
O10^viii^—Ba2—Ba1	139.09 (6)	O11^x^—I6—O12	89.72 (12)
O10^ix^—Ba2—Ba1	139.09 (6)	O11^vii^—I6—O14^xi^	87.84 (11)
O10^viii^—Ba2—Ba3	46.29 (6)	O11^x^—I6—O14^xi^	87.84 (11)
O10^ix^—Ba2—Ba3	46.29 (6)	O12—I6—Ba1^xix^	129.02 (12)
O10^ix^—Ba2—Ba5^viii^	110.75 (6)	O12—I6—Ba1^xvi^	125.337 (9)
O10^viii^—Ba2—Ba5^vii^	110.75 (6)	O12—I6—Ba1^xviii^	125.337 (9)
O10^ix^—Ba2—Ba5^vii^	53.61 (7)	O12—I6—Ba3	48.52 (12)
O10^viii^—Ba2—Ba5^viii^	53.61 (7)	O12—I6—Ba5^vii^	55.336 (10)
O10^ix^—Ba2—I7	99.96 (7)	O12—I6—Ba5^viii^	55.336 (10)
O10^viii^—Ba2—I7	99.96 (7)	O14^xi^—I6—Ba1^xviii^	54.531 (6)
O10^viii^—Ba2—O8^i^	98.76 (9)	O14^xi^—I6—Ba1^xvi^	54.531 (6)
O10^ix^—Ba2—O8^i^	155.68 (8)	O14^xi^—I6—Ba1^xix^	47.47 (11)
O10^ix^—Ba2—O8	98.76 (9)	O14^xi^—I6—Ba3	127.97 (11)
O10^viii^—Ba2—O8	155.68 (8)	O14^xi^—I6—Ba5^viii^	124.833 (6)
O10^ix^—Ba2—O9^i^	86.50 (9)	O14^xi^—I6—Ba5^vii^	124.833 (6)
O10^viii^—Ba2—O9	86.50 (9)	O14^xi^—I6—O12	176.49 (17)
O10^viii^—Ba2—O9^i^	124.45 (9)	Ba2—I7—Ba4^vi^	93.860 (7)
O10^ix^—Ba2—O9	124.45 (9)	Ba2—I7—Ba4^ii^	93.860 (7)
O10^ix^—Ba2—O10^viii^	77.86 (13)	Ba2—I7—Ba5	125.013 (10)
O10^viii^—Ba2—O12	76.60 (9)	Ba3^vi^—I7—Ba2	80.642 (7)
O10^ix^—Ba2—O12	76.60 (9)	Ba3^ii^—I7—Ba2	80.642 (7)
O12—Ba2—Ba1	121.05 (8)	Ba3^ii^—I7—Ba3^vi^	114.195 (11)
O12—Ba2—Ba3	45.32 (8)	Ba3^vi^—I7—Ba4^ii^	173.462 (11)
O12—Ba2—Ba5^vii^	49.054 (7)	Ba3^ii^—I7—Ba4^vi^	173.462 (11)
O12—Ba2—Ba5^viii^	49.054 (7)	Ba3^ii^—I7—Ba4^ii^	67.997 (6)
O12—Ba2—I7	175.50 (8)	Ba3^vi^—I7—Ba4^vi^	67.997 (6)
I7^viii^—Ba3—I7^vii^	114.196 (11)	Ba3^vi^—I7—Ba5	70.658 (7)
O9^ix^—Ba3—I7^vii^	32.11 (5)	Ba3^ii^—I7—Ba5	70.658 (7)
O9^viii^—Ba3—I7^viii^	32.11 (5)	Ba4^vi^—I7—Ba4^ii^	109.129 (11)
O9^viii^—Ba3—I7^vii^	96.85 (5)	Ba4^ii^—I7—Ba5	115.660 (7)
O9^ix^—Ba3—I7^viii^	96.85 (5)	Ba4^vi^—I7—Ba5	115.660 (7)
O9^ix^—Ba3—O9^viii^	70.27 (10)	O9^i^—I7—Ba2	47.57 (8)
O9^ix^—Ba3—O10^ix^	55.24 (7)	O9—I7—Ba2	47.57 (8)
O9^viii^—Ba3—O10^viii^	55.24 (7)	O9^i^—I7—Ba3^ii^	52.75 (8)
O9^ix^—Ba3—O10^viii^	94.55 (8)	O9—I7—Ba3^vi^	52.75 (8)
O9^viii^—Ba3—O10^ix^	94.55 (8)	O9^i^—I7—Ba3^vi^	126.11 (8)
O9^ix^—Ba3—O11^x^	169.96 (7)	O9—I7—Ba3^ii^	126.11 (8)
O9^viii^—Ba3—O11^vii^	169.96 (7)	O9^i^—I7—Ba4^ii^	49.66 (8)
O9^ix^—Ba3—O11^vii^	119.07 (7)	O9^i^—I7—Ba4^vi^	120.80 (9)
O9^viii^—Ba3—O11^x^	119.07 (7)	O9—I7—Ba4^ii^	120.80 (9)
O9^ix^—Ba3—O12	125.79 (7)	O9—I7—Ba4^vi^	49.66 (8)
O9^viii^—Ba3—O12	125.79 (7)	O9—I7—Ba5	123.22 (9)
O9^viii^—Ba3—O13^viii^	55.27 (9)	O9^i^—I7—Ba5	123.22 (9)
O9^ix^—Ba3—O13^viii^	125.51 (9)	O9^i^—I7—O9	90.71 (16)
O9^ix^—Ba3—O13^vii^	55.27 (9)	O9^i^—I7—O13	90.74 (13)
O9^viii^—Ba3—O13^vii^	125.51 (9)	O9—I7—O13	90.74 (13)
O10^viii^—Ba3—I7^viii^	32.12 (5)	O10—I7—Ba2	132.56 (9)
O10^viii^—Ba3—I7^vii^	95.77 (6)	O10^i^—I7—Ba2	132.56 (9)
O10^ix^—Ba3—I7^vii^	32.12 (5)	O10—I7—Ba3^vi^	54.32 (9)
O10^ix^—Ba3—I7^viii^	95.77 (6)	O10^i^—I7—Ba3^ii^	54.32 (9)
O10^viii^—Ba3—O10^ix^	68.70 (11)	O10—I7—Ba3^ii^	128.00 (9)
O10^viii^—Ba3—O11^vii^	124.17 (7)	O10^i^—I7—Ba3^vi^	128.00 (9)
O10^ix^—Ba3—O11^x^	124.17 (7)	O10—I7—Ba4^ii^	129.82 (9)
O10^viii^—Ba3—O11^x^	94.17 (8)	O10^i^—I7—Ba4^vi^	129.82 (9)
O10^ix^—Ba3—O11^vii^	94.17 (8)	O10^i^—I7—Ba4^ii^	58.46 (9)
O10^viii^—Ba3—O13^vii^	123.11 (10)	O10—I7—Ba4^vi^	58.46 (9)
O10^ix^—Ba3—O13^vii^	54.42 (9)	O10^i^—I7—Ba5	57.57 (9)
O10^viii^—Ba3—O13^viii^	54.42 (9)	O10—I7—Ba5	57.57 (9)
O10^ix^—Ba3—O13^viii^	123.11 (10)	O10—I7—O9	89.07 (12)
O11^x^—Ba3—I7^viii^	93.18 (5)	O10^i^—I7—O9	179.10 (13)
O11^x^—Ba3—I7^vii^	141.54 (4)	O10^i^—I7—O9^i^	89.08 (12)
O11^vii^—Ba3—I7^viii^	141.54 (4)	O10—I7—O9^i^	179.10 (13)
O11^vii^—Ba3—I7^vii^	93.18 (5)	O10—I7—O10^i^	91.12 (17)
O11^x^—Ba3—O11^vii^	51.36 (9)	O10—I7—O13	90.13 (13)
O12—Ba3—I7^viii^	95.76 (4)	O10^i^—I7—O13	90.13 (13)
O12—Ba3—I7^vii^	95.76 (4)	O13—I7—Ba2	74.83 (13)
O12—Ba3—O10^viii^	71.16 (8)	O13—I7—Ba3^ii^	57.126 (7)
O12—Ba3—O10^ix^	71.16 (8)	O13—I7—Ba3^vi^	57.126 (7)
O12—Ba3—O11^x^	53.13 (8)	O13—I7—Ba4^ii^	124.989 (15)
O12—Ba3—O11^vii^	53.13 (8)	O13—I7—Ba4^vi^	124.989 (15)
O12—Ba3—O13^vii^	88.69 (9)	O13—I7—Ba5	50.18 (13)
O12—Ba3—O13^viii^	88.69 (9)	O15^iv^—I7—Ba2	102.78 (12)
O13^viii^—Ba3—I7^viii^	32.40 (7)	O15^iv^—I7—Ba3^vi^	122.781 (9)
O13^vii^—Ba3—I7^vii^	32.40 (7)	O15^iv^—I7—Ba3^ii^	122.781 (9)
O13^vii^—Ba3—I7^viii^	146.54 (7)	O15^iv^—I7—Ba4^ii^	54.796 (11)
O13^viii^—Ba3—I7^vii^	146.54 (7)	O15^iv^—I7—Ba4^vi^	54.796 (11)
O13^viii^—Ba3—O11^vii^	115.38 (9)	O15^iv^—I7—Ba5	132.21 (12)
O13^vii^—Ba3—O11^x^	115.38 (9)	O15^iv^—I7—O9	87.58 (12)
O13^viii^—Ba3—O11^x^	64.04 (9)	O15^iv^—I7—O9^i^	87.58 (12)
O13^vii^—Ba3—O11^vii^	64.04 (9)	O15^iv^—I7—O10^i^	91.54 (13)
O13^viii^—Ba3—O13^vii^	176.94 (17)	O15^iv^—I7—O10	91.54 (13)
O15^xi^—Ba3—I7^vii^	118.70 (3)	O15^iv^—I7—O13	177.61 (18)
O15^xi^—Ba3—I7^viii^	118.70 (3)	Ba1—O8—Ba5^vii^	162.43 (9)
O15^xi^—Ba3—O9^ix^	111.90 (9)	Ba2—O8—Ba1	78.42 (7)
O15^xi^—Ba3—O9^viii^	111.90 (9)	Ba2—O8—Ba5^iv^	100.70 (8)
O15^xi^—Ba3—O10^viii^	145.22 (6)	Ba2—O8—Ba5^vii^	85.11 (7)
O15^xi^—Ba3—O10^ix^	145.22 (6)	Ba5^iv^—O8—Ba1	90.31 (7)
O15^xi^—Ba3—O11^x^	62.30 (9)	Ba5^iv^—O8—Ba5^vii^	98.80 (8)
O15^xi^—Ba3—O11^vii^	62.30 (9)	I6^xvi^—O8—Ba1	91.73 (10)
O15^xi^—Ba3—O12	107.21 (11)	I6^xvi^—O8—Ba2	159.07 (13)
O15^xi^—Ba3—O13^viii^	91.14 (8)	I6^xvi^—O8—Ba5^iv^	97.75 (10)
O15^xi^—Ba3—O13^vii^	91.14 (8)	I6^xvi^—O8—Ba5^vii^	101.80 (10)
I7^viii^—Ba4—I7^vii^	109.128 (11)	Ba2—O9—Ba1	74.80 (6)
O9^viii^—Ba4—I7^viii^	30.70 (5)	Ba2—O9—Ba3^vi^	113.03 (10)
O9^ix^—Ba4—I7^vii^	30.70 (5)	Ba2—O9—Ba4^vi^	146.46 (11)
O9^ix^—Ba4—I7^viii^	94.64 (6)	Ba3^vi^—O9—Ba1	168.60 (10)
O9^viii^—Ba4—I7^vii^	94.64 (6)	Ba4^vi^—O9—Ba1	78.85 (7)
O9^ix^—Ba4—O9^viii^	70.52 (11)	Ba4^vi^—O9—Ba3^vi^	90.73 (7)
O9^viii^—Ba4—O10^viii^	52.77 (7)	I7—O9—Ba1	91.20 (10)
O9^ix^—Ba4—O10^viii^	89.92 (8)	I7—O9—Ba2	101.28 (10)
O9^ix^—Ba4—O10^ix^	52.77 (7)	I7—O9—Ba3^vi^	95.15 (10)
O9^viii^—Ba4—O10^ix^	89.92 (8)	I7—O9—Ba4^vi^	99.64 (10)
O9^viii^—Ba4—O11^i^	104.24 (8)	Ba2^vi^—O10—Ba3^vi^	93.24 (8)
O9^ix^—Ba4—O11^i^	152.12 (8)	Ba2^vi^—O10—Ba4^vi^	92.53 (8)
O9^viii^—Ba4—O11	152.12 (8)	Ba2^vi^—O10—Ba5	85.52 (8)
O9^ix^—Ba4—O11	104.24 (8)	Ba3^vi^—O10—Ba4^vi^	84.22 (7)
O9^ix^—Ba4—O15^xiii^	123.37 (9)	Ba3^vi^—O10—Ba5	88.32 (8)
O9^viii^—Ba4—O15^xii^	123.37 (9)	Ba4^vi^—O10—Ba5	172.17 (10)
O9^viii^—Ba4—O15^xiii^	53.26 (9)	I7—O10—Ba2^vi^	172.83 (15)
O9^ix^—Ba4—O15^xii^	53.26 (9)	I7—O10—Ba3^vi^	93.56 (10)
O10^ix^—Ba4—I7^viii^	89.77 (5)	I7—O10—Ba4^vi^	90.41 (10)
O10^ix^—Ba4—I7^vii^	31.13 (5)	I7—O10—Ba5	92.41 (11)
O10^viii^—Ba4—I7^vii^	89.77 (5)	Ba1^xiv^—O11—Ba1^vii^	83.74 (7)
O10^viii^—Ba4—I7^viii^	31.13 (5)	Ba1^vii^—O11—Ba3^ii^	99.37 (7)
O10^viii^—Ba4—O10^ix^	63.04 (10)	Ba1^xiv^—O11—Ba3^ii^	171.81 (9)
O11^i^—Ba4—I7^vii^	158.97 (5)	Ba1^xiv^—O11—Ba4	91.24 (7)
O11^i^—Ba4—I7^viii^	91.89 (5)	Ba1^xiv^—O11—Ba5	90.09 (7)
O11—Ba4—I7^vii^	91.89 (5)	Ba4—O11—Ba1^vii^	79.99 (7)
O11—Ba4—I7^viii^	158.97 (5)	Ba4—O11—Ba3^ii^	81.91 (6)
O11^i^—Ba4—O10^ix^	154.47 (7)	Ba4—O11—Ba5	97.58 (7)
O11—Ba4—O10^viii^	154.47 (7)	Ba5—O11—Ba1^vii^	173.30 (10)
O11^i^—Ba4—O10^viii^	108.94 (7)	Ba5—O11—Ba3^ii^	86.40 (7)
O11—Ba4—O10^ix^	108.94 (7)	I6^ii^—O11—Ba1^xiv^	96.78 (10)
O11—Ba4—O11^i^	67.08 (10)	I6^ii^—O11—Ba1^vii^	91.52 (9)
O11—Ba4—O15^xiii^	128.10 (9)	I6^ii^—O11—Ba3^ii^	90.73 (9)
O11^i^—Ba4—O15^xiii^	61.20 (9)	I6^ii^—O11—Ba4	167.64 (13)
O11^i^—Ba4—O15^xii^	128.10 (9)	I6^ii^—O11—Ba5	91.82 (10)
O11—Ba4—O15^xii^	61.20 (9)	Ba2—O12—Ba3	90.58 (11)
O13—Ba4—I7^vii^	102.72 (5)	Ba2—O12—Ba5^viii^	86.42 (7)
O13—Ba4—I7^viii^	102.72 (5)	Ba2—O12—Ba5^vii^	86.42 (7)
O13—Ba4—O9^ix^	133.19 (8)	Ba3—O12—Ba5^viii^	93.34 (8)
O13—Ba4—O9^viii^	133.19 (8)	Ba3—O12—Ba5^vii^	93.34 (8)
O13—Ba4—O10^ix^	83.85 (9)	Ba5^viii^—O12—Ba5^vii^	170.24 (15)
O13—Ba4—O10^viii^	83.85 (9)	I6—O12—Ba2	168.4 (2)
O13—Ba4—O11^i^	70.94 (9)	I6—O12—Ba3	101.07 (15)
O13—Ba4—O11	70.94 (9)	I6—O12—Ba5^viii^	92.84 (7)
O13—Ba4—O15^xii^	88.24 (8)	I6—O12—Ba5^vii^	92.84 (7)
O13—Ba4—O15^xiii^	88.24 (8)	Ba3^vi^—O13—Ba3^ii^	176.94 (17)
O14—Ba4—I7^viii^	100.50 (5)	Ba4—O13—Ba3^ii^	88.97 (8)
O14—Ba4—I7^vii^	100.50 (5)	Ba4—O13—Ba3^vi^	88.97 (8)
O14—Ba4—O9^viii^	76.73 (8)	Ba4—O13—Ba5	104.54 (13)
O14—Ba4—O9^ix^	76.73 (8)	Ba5—O13—Ba3^ii^	91.35 (9)
O14—Ba4—O10^ix^	129.20 (8)	Ba5—O13—Ba3^vi^	91.35 (9)
O14—Ba4—O10^viii^	129.20 (8)	I7—O13—Ba3^ii^	90.47 (7)
O14—Ba4—O11	75.43 (9)	I7—O13—Ba3^vi^	90.47 (7)
O14—Ba4—O11^i^	75.43 (9)	I7—O13—Ba4	155.4 (2)
O14—Ba4—O13	139.37 (12)	I7—O13—Ba5	100.10 (16)
O14—Ba4—O15^xii^	95.13 (8)	Ba1^xiv^—O14—Ba1^vii^	88.26 (7)
O14—Ba4—O15^xiii^	95.13 (8)	Ba1^xiv^—O14—Ba1^viii^	88.26 (7)
O15^xiii^—Ba4—I7^viii^	30.93 (7)	Ba1^viii^—O14—Ba1^vii^	171.78 (14)
O15^xii^—Ba4—I7^vii^	30.93 (7)	Ba4—O14—Ba1^vii^	86.60 (7)
O15^xii^—Ba4—I7^viii^	139.69 (7)	Ba4—O14—Ba1^xiv^	99.15 (12)
O15^xiii^—Ba4—I7^vii^	139.69 (7)	Ba4—O14—Ba1^viii^	86.60 (7)
O15^xiii^—Ba4—O10^viii^	52.49 (9)	I6^xx^—O14—Ba1^viii^	94.00 (7)
O15^xii^—Ba4—O10^viii^	115.52 (9)	I6^xx^—O14—Ba1^vii^	94.00 (7)
O15^xiii^—Ba4—O10^ix^	115.52 (9)	I6^xx^—O14—Ba1^xiv^	102.20 (15)
O15^xii^—Ba4—O10^ix^	52.49 (9)	I6^xx^—O14—Ba4	158.65 (19)
O15^xiii^—Ba4—O15^xii^	167.84 (15)	Ba1^xiv^—O15—Ba4^xiii^	84.56 (8)
O8^ii^—Ba5—I6^ii^	89.25 (5)	Ba1^xiv^—O15—Ba4^xii^	84.56 (8)
O8^xiv^—Ba5—I6^ii^	31.23 (5)	Ba3^xx^—O15—Ba1^xiv^	127.86 (15)
O8^xv^—Ba5—I6^ii^	94.55 (5)	Ba3^xx^—O15—Ba4^xii^	91.20 (8)
O8^iii^—Ba5—I6^ii^	134.22 (5)	Ba3^xx^—O15—Ba4^xiii^	91.20 (8)
O8^xiv^—Ba5—O8^ii^	81.20 (8)	Ba4^xiii^—O15—Ba4^xii^	167.84 (15)
O8^xiv^—Ba5—O8^iii^	109.88 (5)	I7^xiv^—O15—Ba1^xiv^	108.24 (17)
O8^xv^—Ba5—O8^iii^	81.20 (8)	I7^xiv^—O15—Ba3^xx^	123.91 (19)
O8^xv^—Ba5—O8^ii^	109.88 (5)	I7^xiv^—O15—Ba4^xii^	94.27 (7)
O8^iii^—Ba5—O8^ii^	51.30 (9)	I7^xiv^—O15—Ba4^xiii^	94.27 (7)
O8^xiv^—Ba5—O8^xv^	69.41 (10)		
			
Ba1^xviii^—I6—O12—Ba2	93.41 (9)	Ba5—I7—O9—Ba1	−175.958 (13)
Ba1^xix^—I6—O12—Ba2	180.000 (1)	Ba5—I7—O9—Ba2	109.30 (8)
Ba1^xvi^—I6—O12—Ba2	−93.41 (9)	Ba5—I7—O9—Ba3^vi^	−5.44 (11)
Ba1^xvi^—I6—O12—Ba3	86.59 (9)	Ba5—I7—O9—Ba4^vi^	−97.06 (9)
Ba1^xviii^—I6—O12—Ba3	−86.59 (9)	Ba5—I7—O10—Ba3^vi^	88.46 (9)
Ba1^xix^—I6—O12—Ba3	0.000 (1)	Ba5—I7—O10—Ba4^vi^	172.70 (11)
Ba1^xix^—I6—O12—Ba5^vii^	−93.97 (8)	Ba5—I7—O13—Ba3^ii^	91.45 (9)
Ba1^xviii^—I6—O12—Ba5^vii^	179.445 (14)	Ba5—I7—O13—Ba3^vi^	−91.45 (9)
Ba1^xvi^—I6—O12—Ba5^vii^	−7.38 (17)	Ba5—I7—O13—Ba4	180.000 (1)
Ba1^xvi^—I6—O12—Ba5^viii^	−179.445 (14)	O8^xvi^—I6—O12—Ba2	−45.60 (8)
Ba1^xix^—I6—O12—Ba5^viii^	93.97 (8)	O8^xvii^—I6—O12—Ba2	45.60 (8)
Ba1^xviii^—I6—O12—Ba5^viii^	7.38 (17)	O8^xvi^—I6—O12—Ba3	134.40 (8)
Ba2—I7—O9—Ba1	74.74 (8)	O8^xvii^—I6—O12—Ba3	−134.40 (8)
Ba2—I7—O9—Ba3^vi^	−114.74 (12)	O8^xvi^—I6—O12—Ba5^viii^	−131.63 (12)
Ba2—I7—O9—Ba4^vi^	153.64 (15)	O8^xvii^—I6—O12—Ba5^viii^	−40.43 (12)
Ba2—I7—O10—Ba3^vi^	−21.39 (15)	O8^xvi^—I6—O12—Ba5^vii^	40.43 (12)
Ba2—I7—O10—Ba4^vi^	62.85 (12)	O8^xvii^—I6—O12—Ba5^vii^	131.63 (12)
Ba2—I7—O10—Ba5	−109.85 (8)	O9^i^—I7—O9—Ba1	52.50 (12)
Ba2—I7—O13—Ba3^ii^	−88.55 (9)	O9^i^—I7—O9—Ba2	−22.24 (16)
Ba2—I7—O13—Ba3^vi^	88.55 (9)	O9^i^—I7—O9—Ba3^vi^	−136.98 (6)
Ba2—I7—O13—Ba4	0.0	O9^i^—I7—O9—Ba4^vi^	131.40 (6)
Ba2—I7—O13—Ba5	180.000 (1)	O9—I7—O10—Ba3^vi^	−42.80 (11)
Ba3—I6—O12—Ba2	180.000 (2)	O9—I7—O10—Ba4^vi^	41.44 (10)
Ba3—I6—O12—Ba5^vii^	−93.97 (8)	O9—I7—O10—Ba5	−131.26 (10)
Ba3—I6—O12—Ba5^viii^	93.97 (8)	O9^i^—I7—O13—Ba3^vi^	133.91 (12)
Ba3^vi^—I7—O9—Ba1	−170.52 (11)	O9—I7—O13—Ba3^ii^	−133.91 (12)
Ba3^ii^—I7—O9—Ba1	94.75 (8)	O9—I7—O13—Ba3^vi^	43.19 (12)
Ba3^ii^—I7—O9—Ba2	20.00 (14)	O9^i^—I7—O13—Ba3^ii^	−43.19 (12)
Ba3^vi^—I7—O9—Ba2	114.74 (12)	O9—I7—O13—Ba4	−45.36 (8)
Ba3^ii^—I7—O9—Ba3^vi^	−94.73 (8)	O9^i^—I7—O13—Ba4	45.36 (8)
Ba3^ii^—I7—O9—Ba4^vi^	173.643 (17)	O9^i^—I7—O13—Ba5	−134.64 (8)
Ba3^vi^—I7—O9—Ba4^vi^	−91.62 (9)	O9—I7—O13—Ba5	134.64 (8)
Ba3^ii^—I7—O10—Ba3^vi^	94.55 (9)	O10—I7—O9—Ba1	−126.63 (10)
Ba3^ii^—I7—O10—Ba4^vi^	178.780 (19)	O10—I7—O9—Ba2	158.63 (12)
Ba3^vi^—I7—O10—Ba4^vi^	84.23 (8)	O10—I7—O9—Ba3^vi^	43.89 (11)
Ba3^ii^—I7—O10—Ba5	6.08 (12)	O10—I7—O9—Ba4^vi^	−47.73 (12)
Ba3^vi^—I7—O10—Ba5	−88.46 (9)	O10^i^—I7—O10—Ba3^vi^	138.08 (6)
Ba3^vi^—I7—O13—Ba3^ii^	−177.09 (17)	O10^i^—I7—O10—Ba4^vi^	−137.69 (7)
Ba3^ii^—I7—O13—Ba3^vi^	177.09 (17)	O10^i^—I7—O10—Ba5	49.61 (14)
Ba3^ii^—I7—O13—Ba4	88.55 (9)	O10—I7—O13—Ba3^vi^	−45.89 (12)
Ba3^vi^—I7—O13—Ba4	−88.55 (9)	O10—I7—O13—Ba3^ii^	137.02 (12)
Ba3^vi^—I7—O13—Ba5	91.45 (9)	O10^i^—I7—O13—Ba3^ii^	45.89 (12)
Ba3^ii^—I7—O13—Ba5	−91.45 (9)	O10^i^—I7—O13—Ba3^vi^	−137.02 (12)
Ba4^ii^—I7—O9—Ba1	10.77 (10)	O10^i^—I7—O13—Ba4	134.44 (9)
Ba4^vi^—I7—O9—Ba1	−78.90 (8)	O10—I7—O13—Ba4	−134.44 (9)
Ba4^vi^—I7—O9—Ba2	−153.64 (15)	O10—I7—O13—Ba5	45.56 (9)
Ba4^ii^—I7—O9—Ba2	−63.97 (11)	O10^i^—I7—O13—Ba5	−45.56 (9)
Ba4^ii^—I7—O9—Ba3^vi^	−178.708 (17)	O11^vii^—I6—O12—Ba2	−134.11 (8)
Ba4^vi^—I7—O9—Ba3^vi^	91.62 (9)	O11^x^—I6—O12—Ba2	134.11 (8)
Ba4^ii^—I7—O9—Ba4^vi^	89.67 (8)	O11^vii^—I6—O12—Ba3	45.89 (8)
Ba4^ii^—I7—O10—Ba3^vi^	−173.595 (18)	O11^x^—I6—O12—Ba3	−45.89 (8)
Ba4^vi^—I7—O10—Ba3^vi^	−84.23 (8)	O11^vii^—I6—O12—Ba5^viii^	139.85 (11)
Ba4^ii^—I7—O10—Ba4^vi^	−89.36 (9)	O11^vii^—I6—O12—Ba5^vii^	−48.08 (11)
Ba4^vi^—I7—O10—Ba5	−172.70 (11)	O11^x^—I6—O12—Ba5^viii^	48.08 (11)
Ba4^ii^—I7—O10—Ba5	97.94 (9)	O11^x^—I6—O12—Ba5^vii^	−139.85 (11)
Ba4^vi^—I7—O13—Ba3^vi^	4.55 (18)	O13—I7—O9—Ba1	143.25 (11)
Ba4^vi^—I7—O13—Ba3^ii^	−172.541 (16)	O13—I7—O9—Ba2	68.51 (12)
Ba4^ii^—I7—O13—Ba3^ii^	−4.55 (18)	O13—I7—O9—Ba3^vi^	−46.23 (11)
Ba4^ii^—I7—O13—Ba3^vi^	172.541 (16)	O13—I7—O9—Ba4^vi^	−137.85 (12)
Ba4^vi^—I7—O13—Ba4	−83.99 (9)	O13—I7—O10—Ba3^vi^	47.94 (12)
Ba4^ii^—I7—O13—Ba4	83.99 (9)	O13—I7—O10—Ba4^vi^	132.17 (11)
Ba4^ii^—I7—O13—Ba5	−96.01 (9)	O13—I7—O10—Ba5	−40.52 (11)
Ba4^vi^—I7—O13—Ba5	96.01 (9)	O15^iv^—I7—O9—Ba1	−35.05 (10)
Ba5^vii^—I6—O12—Ba2	−86.03 (8)	O15^iv^—I7—O9—Ba2	−109.79 (12)
Ba5^viii^—I6—O12—Ba2	86.03 (8)	O15^iv^—I7—O9—Ba3^vi^	135.47 (11)
Ba5^viii^—I6—O12—Ba3	−93.97 (8)	O15^iv^—I7—O9—Ba4^vi^	43.85 (11)
Ba5^vii^—I6—O12—Ba3	93.97 (8)	O15^iv^—I7—O10—Ba3^vi^	−130.35 (11)
Ba5^viii^—I6—O12—Ba5^vii^	172.07 (17)	O15^iv^—I7—O10—Ba4^vi^	−46.12 (10)
Ba5^vii^—I6—O12—Ba5^viii^	−172.07 (17)	O15^iv^—I7—O10—Ba5	141.19 (10)

Symmetry codes: (i) *x*, −*y*+1/2, *z*; (ii) −*x*+1/2, −*y*+1, *z*−1/2; (iii) −*x*+1/2, *y*−1/2, *z*−1/2; (iv) *x*−1/2, *y*, −*z*+1/2; (v) *x*−1/2, −*y*+1/2, −*z*+1/2; (vi) −*x*+1/2, −*y*, *z*−1/2; (vii) −*x*+1/2, −*y*+1, *z*+1/2; (viii) −*x*+1/2, −*y*, *z*+1/2; (ix) −*x*+1/2, *y*+1/2, *z*+1/2; (x) −*x*+1/2, *y*−1/2, *z*+1/2; (xi) *x*−1/2, *y*, −*z*+3/2; (xii) −*x*+1, −*y*+1, −*z*+1; (xiii) −*x*+1, −*y*, −*z*+1; (xiv) *x*+1/2, *y*, −*z*+1/2; (xv) *x*+1/2, −*y*+1/2, −*z*+1/2; (xvi) −*x*, −*y*+1, −*z*+1; (xvii) −*x*, *y*−1/2, −*z*+1; (xviii) −*x*, −*y*, −*z*+1; (xix) *x*, *y*, *z*+1; (xx) *x*+1/2, *y*, −*z*+3/2.

### (80K) Crystal data

**Table:**

Ba_5_I_2_O_12_	*D*_x_ = 6.132 Mg m^−^^3^
*M**_r_* = 1132.50	Mo *K*α radiation, λ = 0.71073 Å
Orthorhombic, *P**n**m**a*	Cell parameters from 13815 reflections
*a* = 19.7361 (3) Å	θ = 1.9–36.2°
*b* = 5.8779 (1) Å	µ = 20.90 mm^−^^1^
*c* = 10.5749 (1) Å	*T* = 80 K
*V* = 1226.76 (3) Å^3^	Prism, brown
*Z* = 4	0.22 × 0.12 × 0.08 mm
*F*(000) = 1928	

### (80K) Data collection

**Table:**

XtaLAB Synergy-I with HyPix3000 diffractometer	2462 reflections with *I* > 2σ(*I*)
Detector resolution: 10.0000 pixels mm^-1^	*R*_int_ = 0.033
ω scans	θ_max_ = 33.1°, θ_min_ = 2.1°
Absorption correction: gaussian (CrysAlisPro; Rigaku OD, 2020)	*h* = −27→30
*T*_min_ = 0.150, *T*_max_ = 0.750	*k* = −8→9
16187 measured reflections	*l* = −16→14
2529 independent reflections	

### (80K) Refinement

**Table:**

Refinement on *F*^2^	0 restraints
Least-squares matrix: full	*w* = 1/[σ^2^(*F*_o_^2^) + (0.0092*P*)^2^ + 7.6239*P*] where *P* = (*F*_o_^2^ + 2*F*_c_^2^)/3
*R*[*F*^2^ > 2σ(*F*^2^)] = 0.022	(Δ/σ)_max_ = 0.001
*wR*(*F*^2^) = 0.043	Δρ_max_ = 1.90 e Å^−^^3^
*S* = 1.25	Δρ_min_ = −1.60 e Å^−^^3^
2529 reflections	Extinction correction: SHELXL2018/3 (Sheldrick 2015b), Fc^*^=kFc[1+0.001xFc^2^λ^3^/sin(2θ)]^-1/4^
104 parameters	Extinction coefficient: 0.00060 (2)

### (80K) Special details

**Table:**

Geometry. All esds (except the esd in the dihedral angle between two l.s. planes) are estimated using the full covariance matrix. The cell esds are taken into account individually in the estimation of esds in distances, angles and torsion angles; correlations between esds in cell parameters are only used when they are defined by crystal symmetry. An approximate (isotropic) treatment of cell esds is used for estimating esds involving l.s. planes.

### (80K) Fractional atomic coordinates and isotropic or equivalent isotropic displacement parameters (Å^2^)

**Table:**

	*x*	*y*	*z*	*U*_iso_*/*U*_eq_	
Ba1	0.04800 (2)	0.250000	0.08951 (3)	0.00409 (6)	
Ba2	0.14082 (2)	0.250000	0.38331 (3)	0.00342 (6)	
Ba3	0.19966 (2)	0.250000	0.74015 (3)	0.00461 (6)	
Ba4	0.34267 (2)	0.250000	0.47535 (3)	0.00355 (6)	
Ba5	0.42748 (2)	0.250000	0.08947 (3)	0.00448 (6)	
I6	0.01359 (2)	0.250000	0.74889 (3)	0.00249 (6)	
I7	0.23871 (2)	0.250000	0.10166 (3)	0.00307 (6)	
O8	0.03691 (13)	0.5213 (4)	0.3330 (2)	0.0056 (5)	
O9	0.18812 (14)	0.0229 (5)	0.1838 (2)	0.0074 (5)	
O10	0.28798 (14)	0.0228 (5)	0.0181 (3)	0.0083 (5)	
O11	0.43517 (13)	0.5187 (5)	0.3288 (3)	0.0067 (5)	
O12	0.07588 (19)	0.250000	0.6120 (4)	0.0062 (7)	
O13	0.3025 (2)	0.250000	0.2342 (4)	0.0079 (7)	
O14	0.45602 (18)	0.250000	0.6070 (3)	0.0048 (6)	
O15	0.67265 (19)	0.250000	0.5245 (4)	0.0081 (7)	

### (80K) Atomic displacement parameters (Å^2^)

**Table:**

	*U*^11^	*U*^22^	*U*^33^	*U*^12^	*U*^13^	*U*^23^
Ba1	0.00457 (12)	0.00427 (12)	0.00343 (11)	0.000	0.00003 (9)	0.000
Ba2	0.00324 (11)	0.00415 (12)	0.00288 (11)	0.000	−0.00054 (9)	0.000
Ba3	0.00566 (12)	0.00425 (12)	0.00394 (12)	0.000	0.00160 (9)	0.000
Ba4	0.00339 (11)	0.00438 (12)	0.00288 (11)	0.000	−0.00051 (9)	0.000
Ba5	0.00447 (12)	0.00409 (12)	0.00487 (12)	0.000	−0.00101 (9)	0.000
I6	0.00238 (12)	0.00310 (13)	0.00198 (12)	0.000	0.00006 (9)	0.000
I7	0.00310 (12)	0.00381 (13)	0.00229 (12)	0.000	0.00011 (10)	0.000
O8	0.0048 (10)	0.0055 (12)	0.0065 (11)	0.0025 (9)	−0.0006 (9)	0.0025 (9)
O9	0.0106 (12)	0.0054 (12)	0.0063 (11)	−0.0019 (10)	0.0028 (9)	0.0004 (9)
O10	0.0112 (12)	0.0053 (12)	0.0084 (11)	0.0030 (10)	0.0031 (10)	−0.0012 (9)
O11	0.0065 (11)	0.0059 (11)	0.0077 (11)	−0.0022 (9)	0.0013 (9)	0.0025 (9)
O12	0.0051 (15)	0.0077 (17)	0.0056 (15)	0.000	0.0047 (13)	0.000
O13	0.0104 (17)	0.0101 (17)	0.0033 (15)	0.000	−0.0040 (13)	0.000
O14	0.0044 (14)	0.0063 (16)	0.0038 (15)	0.000	−0.0009 (12)	0.000
O15	0.0065 (15)	0.0112 (18)	0.0066 (16)	0.000	0.0015 (13)	0.000

### (80K) Geometric parameters (Å, º)

**Table:**

Ba1—O8	3.037 (3)	Ba4—I7^viii^	3.6057 (2)
Ba1—O8^i^	3.037 (3)	Ba4—O9^ix^	2.793 (3)
Ba1—O9	3.229 (3)	Ba4—O9^viii^	2.793 (3)
Ba1—O9^i^	3.229 (3)	Ba4—O10^ix^	3.070 (3)
Ba1—O11^ii^	3.092 (3)	Ba4—O10^viii^	3.070 (3)
Ba1—O11^iii^	3.092 (3)	Ba4—O11^i^	2.869 (3)
Ba1—O11^iv^	2.864 (3)	Ba4—O11	2.869 (3)
Ba1—O11^v^	2.864 (3)	Ba4—O13	2.670 (4)
Ba1—O14^ii^	2.9458 (3)	Ba4—O14	2.635 (4)
Ba1—O14^vi^	2.9458 (3)	Ba4—O15^xii^	2.9545 (4)
Ba1—O14^iv^	2.759 (4)	Ba4—O15^xiii^	2.9545 (4)
Ba1—O15^iv^	2.740 (4)	Ba5—I6^ii^	3.5822 (2)
Ba2—Ba3	3.9482 (4)	Ba5—O8^ii^	3.107 (3)
Ba2—Ba5^vii^	3.8997 (3)	Ba5—O8^xiv^	2.807 (3)
Ba2—Ba5^viii^	3.8997 (3)	Ba5—O8^xv^	2.807 (3)
Ba2—I7	3.5501 (4)	Ba5—O8^iii^	3.107 (3)
Ba2—O8^i^	2.652 (3)	Ba5—O10	3.152 (3)
Ba2—O8	2.652 (3)	Ba5—O10^i^	3.152 (3)
Ba2—O9^i^	2.665 (3)	Ba5—O11^i^	2.987 (3)
Ba2—O9	2.665 (3)	Ba5—O11	2.987 (3)
Ba2—O10^viii^	2.565 (3)	Ba5—O12^vi^	2.9494 (3)
Ba2—O10^ix^	2.565 (3)	Ba5—O12^ii^	2.9494 (3)
Ba2—O12	2.737 (4)	Ba5—O13	2.903 (4)
Ba3—I7^viii^	3.5016 (2)	I6—O8^xvi^	1.884 (3)
Ba3—I7^vii^	3.5016 (2)	I6—O8^xvii^	1.884 (3)
Ba3—O9^viii^	2.799 (3)	I6—O11^vii^	1.893 (3)
Ba3—O9^ix^	2.799 (3)	I6—O11^x^	1.893 (3)
Ba3—O10^ix^	2.854 (3)	I6—O12	1.899 (4)
Ba3—O10^viii^	2.854 (3)	I6—O14^xi^	1.901 (4)
Ba3—O11^vii^	3.132 (3)	I7—O9^i^	1.880 (3)
Ba3—O11^x^	3.132 (3)	I7—O9	1.880 (3)
Ba3—O12	2.794 (4)	I7—O10	1.873 (3)
Ba3—O13^vii^	2.9399 (1)	I7—O10^i^	1.873 (3)
Ba3—O13^viii^	2.9399 (1)	I7—O13	1.884 (4)
Ba3—O15^xi^	2.545 (4)	I7—O15^iv^	1.866 (4)
Ba4—I7^vii^	3.6057 (2)		
			
O8^i^—Ba1—O8	63.36 (10)	O8^iii^—Ba5—O10^i^	99.90 (7)
O8^i^—Ba1—O9^i^	90.97 (7)	O8^xv^—Ba5—O10^i^	169.34 (7)
O8—Ba1—O9^i^	65.33 (7)	O8^ii^—Ba5—O10^i^	78.78 (7)
O8^i^—Ba1—O9	65.33 (7)	O8^xiv^—Ba5—O10^i^	120.09 (7)
O8—Ba1—O9	90.97 (7)	O8^iii^—Ba5—O10	78.77 (7)
O8^i^—Ba1—O11^iii^	122.21 (7)	O8^ii^—Ba5—O10	99.90 (7)
O8—Ba1—O11^iii^	174.14 (7)	O8^xv^—Ba5—O10	120.09 (7)
O8^i^—Ba1—O11^ii^	174.14 (7)	O8^xiv^—Ba5—O10	169.35 (7)
O8—Ba1—O11^ii^	122.21 (7)	O8^xiv^—Ba5—O11^i^	90.80 (8)
O9^i^—Ba1—O9	48.85 (10)	O8^xv^—Ba5—O11	90.80 (7)
O11^v^—Ba1—O8^i^	53.02 (7)	O8^xv^—Ba5—O11^i^	54.06 (7)
O11^iv^—Ba1—O8^i^	88.73 (7)	O8^xiv^—Ba5—O11	54.06 (7)
O11^v^—Ba1—O8	88.73 (7)	O8^xiv^—Ba5—O12^ii^	55.08 (9)
O11^iv^—Ba1—O8	53.02 (7)	O8^xv^—Ba5—O12^vi^	55.08 (9)
O11^v^—Ba1—O9^i^	143.22 (7)	O8^xv^—Ba5—O12^ii^	124.04 (9)
O11^v^—Ba1—O9	110.17 (7)	O8^xiv^—Ba5—O12^vi^	124.04 (9)
O11^iii^—Ba1—O9	90.10 (7)	O8^xv^—Ba5—O13	119.99 (8)
O11^iv^—Ba1—O9^i^	110.17 (7)	O8^xiv^—Ba5—O13	119.99 (8)
O11^iv^—Ba1—O9	143.22 (7)	O10—Ba5—I6^ii^	138.07 (5)
O11^iii^—Ba1—O9^i^	111.43 (7)	O10^i^—Ba5—I6^ii^	92.79 (5)
O11^ii^—Ba1—O9	111.43 (7)	O10—Ba5—O10^i^	50.13 (10)
O11^ii^—Ba1—O9^i^	90.10 (7)	O11—Ba5—I6^ii^	31.89 (5)
O11^iii^—Ba1—O11^ii^	52.17 (10)	O11^i^—Ba5—I6^ii^	91.07 (5)
O11^v^—Ba1—O11^iv^	66.95 (10)	O11^i^—Ba5—O8^ii^	163.07 (7)
O11^v^—Ba1—O11^ii^	126.52 (4)	O11—Ba5—O8^ii^	119.95 (7)
O11^v^—Ba1—O11^iii^	96.32 (7)	O11—Ba5—O8^iii^	163.07 (7)
O11^iv^—Ba1—O11^iii^	126.52 (4)	O11^i^—Ba5—O8^iii^	119.95 (7)
O11^iv^—Ba1—O11^ii^	96.32 (7)	O11—Ba5—O10^i^	91.33 (7)
O11^iv^—Ba1—O14^ii^	53.83 (9)	O11^i^—Ba5—O10^i^	118.12 (7)
O11^v^—Ba1—O14^ii^	120.69 (9)	O11—Ba5—O10	118.12 (7)
O11^v^—Ba1—O14^vi^	53.83 (9)	O11^i^—Ba5—O10	91.33 (7)
O11^iv^—Ba1—O14^vi^	120.69 (9)	O11—Ba5—O11^i^	63.85 (10)
O14^iv^—Ba1—O8	126.24 (7)	O12^ii^—Ba5—I6^ii^	31.97 (7)
O14^vi^—Ba1—O8	117.95 (9)	O12^vi^—Ba5—I6^ii^	141.89 (7)
O14^ii^—Ba1—O8	54.61 (9)	O12^ii^—Ba5—O8^ii^	69.18 (9)
O14^ii^—Ba1—O8^i^	117.95 (9)	O12^vi^—Ba5—O8^ii^	120.45 (9)
O14^vi^—Ba1—O8^i^	54.61 (9)	O12^vi^—Ba5—O8^iii^	69.18 (9)
O14^iv^—Ba1—O8^i^	126.24 (7)	O12^ii^—Ba5—O8^iii^	120.45 (9)
O14^iv^—Ba1—O9	142.76 (7)	O12^vi^—Ba5—O10^i^	114.95 (9)
O14^ii^—Ba1—O9^i^	65.87 (9)	O12^ii^—Ba5—O10^i^	65.01 (9)
O14^iv^—Ba1—O9^i^	142.76 (7)	O12^ii^—Ba5—O10	114.95 (9)
O14^ii^—Ba1—O9	114.60 (9)	O12^vi^—Ba5—O10	65.01 (9)
O14^vi^—Ba1—O9	65.87 (9)	O12^vi^—Ba5—O11^i^	53.54 (9)
O14^vi^—Ba1—O9^i^	114.60 (9)	O12^ii^—Ba5—O11	53.54 (9)
O14^iv^—Ba1—O11^iv^	73.48 (8)	O12^vi^—Ba5—O11	117.36 (9)
O14^iv^—Ba1—O11^v^	73.48 (8)	O12^ii^—Ba5—O11^i^	117.36 (9)
O14^vi^—Ba1—O11^ii^	119.83 (9)	O12^ii^—Ba5—O12^vi^	170.37 (15)
O14^iv^—Ba1—O11^iii^	53.07 (8)	O13—Ba5—I6^ii^	91.59 (4)
O14^ii^—Ba1—O11^ii^	67.68 (9)	O13—Ba5—O8^iii^	130.72 (8)
O14^ii^—Ba1—O11^iii^	119.83 (9)	O13—Ba5—O8^ii^	130.72 (8)
O14^iv^—Ba1—O11^ii^	53.07 (8)	O13—Ba5—O10	51.97 (8)
O14^vi^—Ba1—O11^iii^	67.68 (9)	O13—Ba5—O10^i^	51.97 (8)
O14^iv^—Ba1—O14^ii^	91.69 (7)	O13—Ba5—O11^i^	66.20 (8)
O14^iv^—Ba1—O14^vi^	91.69 (7)	O13—Ba5—O11	66.20 (8)
O14^ii^—Ba1—O14^vi^	172.17 (14)	O13—Ba5—O12^ii^	86.46 (8)
O15^iv^—Ba1—O8^i^	115.98 (8)	O13—Ba5—O12^vi^	86.46 (8)
O15^iv^—Ba1—O8	115.98 (8)	Ba1^xvi^—I6—Ba1^xviii^	108.979 (11)
O15^iv^—Ba1—O9^i^	50.72 (8)	Ba1^xvi^—I6—Ba1^xix^	66.249 (7)
O15^iv^—Ba1—O9	50.72 (8)	Ba1^xviii^—I6—Ba1^xix^	66.249 (7)
O15^iv^—Ba1—O11^ii^	60.73 (8)	Ba1^xvi^—I6—Ba3	110.393 (7)
O15^iv^—Ba1—O11^v^	146.21 (5)	Ba1^xix^—I6—Ba3	80.763 (9)
O15^iv^—Ba1—O11^iv^	146.21 (5)	Ba1^xviii^—I6—Ba3	110.393 (7)
O15^iv^—Ba1—O11^iii^	60.73 (8)	Ba5^viii^—I6—Ba1^xvi^	179.206 (10)
O15^iv^—Ba1—O14^iv^	105.02 (11)	Ba5^vii^—I6—Ba1^xviii^	179.206 (10)
O15^iv^—Ba1—O14^ii^	92.97 (7)	Ba5^viii^—I6—Ba1^xviii^	70.380 (5)
O15^iv^—Ba1—O14^vi^	92.97 (7)	Ba5^viii^—I6—Ba1^xix^	113.722 (7)
Ba1—Ba2—Ba3	166.582 (11)	Ba5^vii^—I6—Ba1^xix^	113.722 (7)
Ba1—Ba2—Ba5^vii^	107.819 (7)	Ba5^vii^—I6—Ba1^xvi^	70.380 (5)
Ba1—Ba2—Ba5^viii^	107.819 (7)	Ba5^viii^—I6—Ba3	70.341 (7)
Ba5^viii^—Ba2—Ba3	64.364 (6)	Ba5^vii^—I6—Ba3	70.341 (7)
Ba5^vii^—Ba2—Ba3	64.364 (6)	Ba5^viii^—I6—Ba5^vii^	110.256 (11)
Ba5^vii^—Ba2—Ba5^viii^	97.813 (9)	O8^xvi^—I6—Ba1^xviii^	128.33 (8)
I7—Ba2—Ba1	63.494 (8)	O8^xvi^—I6—Ba1^xix^	123.35 (8)
I7—Ba2—Ba3	129.924 (10)	O8^xvii^—I6—Ba1^xviii^	57.24 (8)
I7—Ba2—Ba5^vii^	131.082 (5)	O8^xvii^—I6—Ba1^xix^	123.35 (8)
I7—Ba2—Ba5^viii^	131.082 (5)	O8^xvii^—I6—Ba1^xvi^	128.33 (8)
O8^i^—Ba2—Ba1	55.56 (6)	O8^xvi^—I6—Ba1^xvi^	57.24 (8)
O8—Ba2—Ba1	55.56 (6)	O8^xvi^—I6—Ba3	121.15 (8)
O8—Ba2—Ba3	114.78 (6)	O8^xvii^—I6—Ba3	121.15 (8)
O8^i^—Ba2—Ba3	114.78 (6)	O8^xvi^—I6—Ba5^vii^	50.96 (8)
O8^i^—Ba2—Ba5^vii^	107.34 (6)	O8^xvii^—I6—Ba5^vii^	122.73 (8)
O8^i^—Ba2—Ba5^viii^	52.52 (6)	O8^xvii^—I6—Ba5^viii^	50.96 (8)
O8—Ba2—Ba5^viii^	107.34 (6)	O8^xvi^—I6—Ba5^viii^	122.73 (8)
O8—Ba2—Ba5^vii^	52.52 (6)	O8^xvii^—I6—O8^xvi^	91.01 (16)
O8^i^—Ba2—I7	104.63 (6)	O8^xvi^—I6—O11^vii^	88.60 (12)
O8—Ba2—I7	104.63 (6)	O8^xvii^—I6—O11^vii^	179.13 (12)
O8^i^—Ba2—O8	73.95 (11)	O8^xvii^—I6—O11^x^	88.60 (12)
O8^i^—Ba2—O9^i^	114.42 (8)	O8^xvi^—I6—O11^x^	179.13 (12)
O8—Ba2—O9^i^	79.10 (8)	O8^xvii^—I6—O12	89.54 (11)
O8^i^—Ba2—O9	79.10 (8)	O8^xvi^—I6—O12	89.54 (11)
O8—Ba2—O9	114.42 (8)	O8^xvii^—I6—O14^xi^	93.01 (11)
O8^i^—Ba2—O12	79.34 (8)	O8^xvi^—I6—O14^xi^	93.01 (11)
O8—Ba2—O12	79.34 (8)	O11^vii^—I6—Ba1^xix^	57.49 (8)
O9^i^—Ba2—Ba1	59.74 (6)	O11^x^—I6—Ba1^xviii^	51.98 (8)
O9—Ba2—Ba1	59.74 (6)	O11^x^—I6—Ba1^xvi^	123.58 (8)
O9^i^—Ba2—Ba3	130.80 (6)	O11^vii^—I6—Ba1^xviii^	123.58 (8)
O9—Ba2—Ba3	130.80 (6)	O11^vii^—I6—Ba1^xvi^	51.98 (8)
O9^i^—Ba2—Ba5^viii^	160.23 (6)	O11^x^—I6—Ba1^xix^	57.49 (8)
O9—Ba2—Ba5^vii^	160.23 (6)	O11^x^—I6—Ba3	58.48 (8)
O9^i^—Ba2—Ba5^vii^	100.72 (6)	O11^vii^—I6—Ba3	58.48 (8)
O9—Ba2—Ba5^viii^	100.72 (6)	O11^vii^—I6—Ba5^viii^	128.73 (8)
O9^i^—Ba2—I7	31.28 (6)	O11^vii^—I6—Ba5^vii^	56.46 (8)
O9—Ba2—I7	31.28 (6)	O11^x^—I6—Ba5^vii^	128.73 (8)
O9^i^—Ba2—O9	60.12 (11)	O11^x^—I6—Ba5^viii^	56.46 (8)
O9^i^—Ba2—O12	149.69 (6)	O11^vii^—I6—O11^x^	91.78 (16)
O9—Ba2—O12	149.69 (6)	O11^x^—I6—O12	89.67 (11)
O10^ix^—Ba2—Ba1	139.20 (6)	O11^vii^—I6—O12	89.67 (12)
O10^viii^—Ba2—Ba1	139.20 (6)	O11^x^—I6—O14^xi^	87.79 (11)
O10^viii^—Ba2—Ba3	46.18 (6)	O11^vii^—I6—O14^xi^	87.79 (11)
O10^ix^—Ba2—Ba3	46.18 (6)	O12—I6—Ba1^xvi^	125.360 (9)
O10^ix^—Ba2—Ba5^viii^	110.48 (6)	O12—I6—Ba1^xix^	128.98 (12)
O10^viii^—Ba2—Ba5^vii^	110.48 (6)	O12—I6—Ba1^xviii^	125.360 (9)
O10^viii^—Ba2—Ba5^viii^	53.66 (6)	O12—I6—Ba3	48.21 (12)
O10^ix^—Ba2—Ba5^vii^	53.66 (6)	O12—I6—Ba5^viii^	55.328 (10)
O10^ix^—Ba2—I7	99.68 (6)	O12—I6—Ba5^vii^	55.328 (10)
O10^viii^—Ba2—I7	99.68 (6)	O12—I6—O14^xi^	176.36 (16)
O10^viii^—Ba2—O8^i^	99.15 (9)	O14^xi^—I6—Ba1^xviii^	54.500 (6)
O10^viii^—Ba2—O8	155.67 (8)	O14^xi^—I6—Ba1^xix^	47.38 (11)
O10^ix^—Ba2—O8	99.15 (9)	O14^xi^—I6—Ba1^xvi^	54.500 (6)
O10^ix^—Ba2—O8^i^	155.67 (8)	O14^xi^—I6—Ba3	128.15 (11)
O10^viii^—Ba2—O9^i^	124.14 (9)	O14^xi^—I6—Ba5^viii^	124.845 (7)
O10^ix^—Ba2—O9^i^	86.26 (9)	O14^xi^—I6—Ba5^vii^	124.845 (6)
O10^viii^—Ba2—O9	86.26 (9)	Ba2—I7—Ba4^vi^	93.922 (8)
O10^ix^—Ba2—O9	124.14 (9)	Ba2—I7—Ba4^ii^	93.922 (8)
O10^ix^—Ba2—O10^viii^	77.40 (12)	Ba2—I7—Ba5	124.951 (11)
O10^ix^—Ba2—O12	76.43 (9)	Ba3^vi^—I7—Ba2	80.685 (8)
O10^viii^—Ba2—O12	76.43 (9)	Ba3^ii^—I7—Ba2	80.685 (8)
O12—Ba2—Ba1	121.56 (8)	Ba3^ii^—I7—Ba3^vi^	114.135 (12)
O12—Ba2—Ba3	45.03 (8)	Ba3^vi^—I7—Ba4^ii^	173.593 (12)
O12—Ba2—Ba5^vii^	49.017 (7)	Ba3^ii^—I7—Ba4^vi^	173.593 (12)
O12—Ba2—Ba5^viii^	49.017 (7)	Ba3^ii^—I7—Ba4^ii^	68.009 (6)
O12—Ba2—I7	174.95 (8)	Ba3^vi^—I7—Ba4^vi^	68.009 (6)
I7^viii^—Ba3—I7^vii^	114.134 (12)	Ba3^vi^—I7—Ba5	70.569 (7)
O9^viii^—Ba3—I7^viii^	32.34 (5)	Ba3^ii^—I7—Ba5	70.569 (7)
O9^ix^—Ba3—I7^viii^	96.73 (6)	Ba4^vi^—I7—Ba4^ii^	109.191 (11)
O9^viii^—Ba3—I7^vii^	96.73 (6)	Ba4^ii^—I7—Ba5	115.616 (7)
O9^ix^—Ba3—I7^vii^	32.34 (5)	Ba4^vi^—I7—Ba5	115.616 (7)
O9^ix^—Ba3—O9^viii^	69.93 (11)	O9—I7—Ba2	47.41 (8)
O9^ix^—Ba3—O10^ix^	55.61 (8)	O9^i^—I7—Ba2	47.41 (8)
O9^viii^—Ba3—O10^viii^	55.61 (8)	O9—I7—Ba3^ii^	125.96 (8)
O9^ix^—Ba3—O10^viii^	94.55 (8)	O9^i^—I7—Ba3^vi^	125.96 (8)
O9^viii^—Ba3—O10^ix^	94.55 (8)	O9—I7—Ba3^vi^	52.78 (8)
O9^viii^—Ba3—O11^x^	119.17 (7)	O9^i^—I7—Ba3^ii^	52.78 (8)
O9^ix^—Ba3—O11^x^	170.03 (8)	O9—I7—Ba4^ii^	120.90 (8)
O9^ix^—Ba3—O11^vii^	119.17 (7)	O9—I7—Ba4^vi^	49.90 (8)
O9^viii^—Ba3—O11^vii^	170.03 (8)	O9^i^—I7—Ba4^ii^	49.90 (8)
O9^ix^—Ba3—O13^vii^	55.53 (9)	O9^i^—I7—Ba4^vi^	120.90 (9)
O9^viii^—Ba3—O13^vii^	125.44 (10)	O9^i^—I7—Ba5	123.14 (8)
O9^ix^—Ba3—O13^viii^	125.44 (10)	O9—I7—Ba5	123.14 (8)
O9^viii^—Ba3—O13^viii^	55.53 (9)	O9—I7—O9^i^	90.50 (16)
O10^viii^—Ba3—I7^vii^	95.62 (6)	O9—I7—O13	90.63 (12)
O10^ix^—Ba3—I7^viii^	95.62 (6)	O9^i^—I7—O13	90.63 (12)
O10^viii^—Ba3—I7^viii^	32.28 (5)	O10^i^—I7—Ba2	132.70 (8)
O10^ix^—Ba3—I7^vii^	32.28 (5)	O10—I7—Ba2	132.70 (8)
O10^viii^—Ba3—O10^ix^	68.38 (11)	O10^i^—I7—Ba3^vi^	127.97 (9)
O10^ix^—Ba3—O11^x^	124.25 (7)	O10—I7—Ba3^ii^	127.97 (9)
O10^ix^—Ba3—O11^vii^	94.30 (7)	O10^i^—I7—Ba3^ii^	54.46 (8)
O10^viii^—Ba3—O11^x^	94.30 (7)	O10—I7—Ba3^vi^	54.46 (8)
O10^viii^—Ba3—O11^vii^	124.25 (7)	O10^i^—I7—Ba4^ii^	58.37 (9)
O10^viii^—Ba3—O13^vii^	123.06 (9)	O10—I7—Ba4^vi^	58.37 (9)
O10^ix^—Ba3—O13^vii^	54.68 (9)	O10—I7—Ba4^ii^	129.60 (8)
O10^viii^—Ba3—O13^viii^	54.68 (9)	O10^i^—I7—Ba4^vi^	129.60 (8)
O10^ix^—Ba3—O13^viii^	123.06 (9)	O10—I7—Ba5	57.65 (9)
O11^vii^—Ba3—I7^viii^	141.69 (5)	O10^i^—I7—Ba5	57.65 (9)
O11^vii^—Ba3—I7^vii^	93.21 (5)	O10^i^—I7—O9^i^	89.28 (12)
O11^x^—Ba3—I7^vii^	141.69 (5)	O10—I7—O9^i^	179.11 (12)
O11^x^—Ba3—I7^viii^	93.21 (5)	O10—I7—O9	89.28 (12)
O11^x^—Ba3—O11^vii^	51.45 (10)	O10^i^—I7—O9	179.11 (12)
O12—Ba3—I7^vii^	95.79 (4)	O10^i^—I7—O10	90.92 (17)
O12—Ba3—I7^viii^	95.79 (4)	O10^i^—I7—O13	90.23 (12)
O12—Ba3—O9^ix^	126.07 (7)	O10—I7—O13	90.23 (12)
O12—Ba3—O9^viii^	126.07 (7)	O13—I7—Ba2	74.90 (12)
O12—Ba3—O10^ix^	71.08 (8)	O13—I7—Ba3^ii^	57.095 (7)
O12—Ba3—O10^viii^	71.08 (8)	O13—I7—Ba3^vi^	57.095 (7)
O12—Ba3—O11^vii^	53.28 (8)	O13—I7—Ba4^ii^	124.977 (14)
O12—Ba3—O11^x^	53.28 (8)	O13—I7—Ba4^vi^	124.977 (14)
O12—Ba3—O13^viii^	88.68 (8)	O13—I7—Ba5	50.05 (12)
O12—Ba3—O13^vii^	88.68 (8)	O15^iv^—I7—Ba2	102.70 (12)
O13^vii^—Ba3—I7^viii^	146.63 (7)	O15^iv^—I7—Ba3^vi^	122.812 (9)
O13^vii^—Ba3—I7^vii^	32.55 (7)	O15^iv^—I7—Ba3^ii^	122.812 (9)
O13^viii^—Ba3—I7^viii^	32.55 (7)	O15^iv^—I7—Ba4^ii^	54.813 (10)
O13^viii^—Ba3—I7^vii^	146.63 (7)	O15^iv^—I7—Ba4^vi^	54.813 (10)
O13^viii^—Ba3—O11^x^	63.90 (9)	O15^iv^—I7—Ba5	132.35 (12)
O13^vii^—Ba3—O11^x^	115.34 (9)	O15^iv^—I7—O9^i^	87.68 (12)
O13^vii^—Ba3—O11^vii^	63.90 (9)	O15^iv^—I7—O9	87.68 (12)
O13^viii^—Ba3—O11^vii^	115.34 (9)	O15^iv^—I7—O10	91.45 (12)
O13^viii^—Ba3—O13^vii^	177.04 (16)	O15^iv^—I7—O10^i^	91.45 (12)
O15^xi^—Ba3—I7^vii^	118.80 (3)	O15^iv^—I7—O13	177.60 (17)
O15^xi^—Ba3—I7^viii^	118.80 (3)	Ba1—O8—Ba5^vii^	161.98 (9)
O15^xi^—Ba3—O9^viii^	111.96 (9)	Ba2—O8—Ba1	78.37 (7)
O15^xi^—Ba3—O9^ix^	111.96 (9)	Ba2—O8—Ba5^iv^	101.23 (8)
O15^xi^—Ba3—O10^ix^	145.32 (6)	Ba2—O8—Ba5^vii^	84.85 (7)
O15^xi^—Ba3—O10^viii^	145.32 (6)	Ba5^iv^—O8—Ba1	90.26 (7)
O15^xi^—Ba3—O11^x^	61.92 (9)	Ba5^iv^—O8—Ba5^vii^	99.50 (8)
O15^xi^—Ba3—O11^vii^	61.92 (9)	I6^xvi^—O8—Ba1	91.31 (9)
O15^xi^—Ba3—O12	106.92 (11)	I6^xvi^—O8—Ba2	158.48 (13)
O15^xi^—Ba3—O13^viii^	91.02 (7)	I6^xvi^—O8—Ba5^iv^	97.62 (10)
O15^xi^—Ba3—O13^vii^	91.02 (7)	I6^xvi^—O8—Ba5^vii^	102.26 (10)
I7^viii^—Ba4—I7^vii^	109.190 (11)	Ba2—O9—Ba1	74.77 (7)
O9^viii^—Ba4—I7^viii^	30.98 (5)	Ba2—O9—Ba3^vi^	113.32 (9)
O9^ix^—Ba4—I7^vii^	30.98 (5)	Ba2—O9—Ba4^vi^	146.73 (11)
O9^ix^—Ba4—I7^viii^	94.51 (6)	Ba3^vi^—O9—Ba1	168.67 (10)
O9^viii^—Ba4—I7^vii^	94.51 (6)	Ba4^vi^—O9—Ba1	78.89 (7)
O9^ix^—Ba4—O9^viii^	70.09 (11)	Ba4^vi^—O9—Ba3^vi^	90.63 (8)
O9^viii^—Ba4—O10^viii^	53.21 (7)	I7—O9—Ba1	91.06 (10)
O9^ix^—Ba4—O10^viii^	90.05 (8)	I7—O9—Ba2	101.31 (11)
O9^ix^—Ba4—O10^ix^	53.21 (7)	I7—O9—Ba3^vi^	94.88 (10)
O9^viii^—Ba4—O10^ix^	90.05 (8)	I7—O9—Ba4^vi^	99.12 (10)
O9^viii^—Ba4—O11^i^	104.40 (8)	Ba2^vi^—O10—Ba3^vi^	93.39 (8)
O9^ix^—Ba4—O11^i^	151.76 (8)	Ba2^vi^—O10—Ba4^vi^	92.96 (8)
O9^viii^—Ba4—O11	151.76 (8)	Ba2^vi^—O10—Ba5	85.38 (7)
O9^ix^—Ba4—O11	104.40 (8)	Ba3^vi^—O10—Ba4^vi^	84.23 (7)
O9^ix^—Ba4—O15^xiii^	123.27 (9)	Ba3^vi^—O10—Ba5	88.08 (7)
O9^viii^—Ba4—O15^xii^	123.27 (9)	Ba4^vi^—O10—Ba5	172.02 (10)
O9^viii^—Ba4—O15^xiii^	53.58 (9)	I7—O10—Ba2^vi^	172.85 (15)
O9^ix^—Ba4—O15^xii^	53.58 (9)	I7—O10—Ba3^vi^	93.26 (10)
O10^ix^—Ba4—I7^viii^	89.83 (5)	I7—O10—Ba4^vi^	90.33 (10)
O10^ix^—Ba4—I7^vii^	31.30 (5)	I7—O10—Ba5	92.21 (10)
O10^viii^—Ba4—I7^vii^	89.83 (5)	Ba1^xiv^—O11—Ba1^vii^	83.68 (7)
O10^viii^—Ba4—I7^viii^	31.30 (5)	Ba1^vii^—O11—Ba3^ii^	99.63 (8)
O10^viii^—Ba4—O10^ix^	62.98 (10)	Ba1^xiv^—O11—Ba3^ii^	172.10 (10)
O11^i^—Ba4—I7^vii^	158.81 (5)	Ba1^xiv^—O11—Ba4	91.61 (8)
O11^i^—Ba4—I7^viii^	92.00 (5)	Ba1^xiv^—O11—Ba5	90.20 (7)
O11—Ba4—I7^vii^	92.00 (5)	Ba4—O11—Ba1^vii^	80.14 (7)
O11—Ba4—I7^viii^	158.81 (5)	Ba4—O11—Ba3^ii^	81.95 (6)
O11^i^—Ba4—O10^ix^	154.38 (7)	Ba4—O11—Ba5	97.72 (8)
O11—Ba4—O10^viii^	154.38 (7)	Ba5—O11—Ba1^vii^	173.43 (10)
O11^i^—Ba4—O10^viii^	109.05 (7)	Ba5—O11—Ba3^ii^	86.15 (7)
O11—Ba4—O10^ix^	109.05 (7)	I6^ii^—O11—Ba1^xiv^	96.62 (10)
O11—Ba4—O11^i^	66.81 (11)	I6^ii^—O11—Ba1^vii^	91.42 (10)
O11—Ba4—O15^xiii^	127.81 (9)	I6^ii^—O11—Ba3^ii^	90.50 (9)
O11^i^—Ba4—O15^xiii^	61.16 (9)	I6^ii^—O11—Ba4	167.51 (13)
O11^i^—Ba4—O15^xii^	127.81 (9)	I6^ii^—O11—Ba5	91.65 (10)
O11—Ba4—O15^xii^	61.16 (9)	Ba2—O12—Ba3	91.09 (11)
O13—Ba4—I7^vii^	102.80 (5)	Ba2—O12—Ba5^viii^	86.51 (7)
O13—Ba4—I7^viii^	102.80 (5)	Ba2—O12—Ba5^vii^	86.51 (7)
O13—Ba4—O9^ix^	133.56 (8)	Ba3—O12—Ba5^viii^	93.38 (7)
O13—Ba4—O9^viii^	133.56 (8)	Ba3—O12—Ba5^vii^	93.38 (7)
O13—Ba4—O10^ix^	83.76 (9)	Ba5^viii^—O12—Ba5^vii^	170.37 (15)
O13—Ba4—O10^viii^	83.76 (9)	I6—O12—Ba2	167.6 (2)
O13—Ba4—O11^i^	70.92 (9)	I6—O12—Ba3	101.33 (15)
O13—Ba4—O11	70.91 (9)	I6—O12—Ba5^viii^	92.70 (7)
O13—Ba4—O15^xii^	88.28 (8)	I6—O12—Ba5^vii^	92.70 (7)
O13—Ba4—O15^xiii^	88.28 (8)	Ba3^vi^—O13—Ba3^ii^	177.04 (16)
O14—Ba4—I7^viii^	100.51 (4)	Ba4—O13—Ba3^ii^	89.08 (7)
O14—Ba4—I7^vii^	100.51 (4)	Ba4—O13—Ba3^vi^	89.08 (7)
O14—Ba4—O9^viii^	76.57 (8)	Ba4—O13—Ba5	104.55 (13)
O14—Ba4—O9^ix^	76.57 (8)	Ba5—O13—Ba3^ii^	91.35 (8)
O14—Ba4—O10^ix^	129.44 (8)	Ba5—O13—Ba3^vi^	91.35 (8)
O14—Ba4—O10^viii^	129.44 (8)	I7—O13—Ba3^ii^	90.35 (7)
O14—Ba4—O11	75.24 (8)	I7—O13—Ba3^vi^	90.35 (7)
O14—Ba4—O11^i^	75.24 (8)	I7—O13—Ba4	155.3 (2)
O14—Ba4—O13	139.17 (12)	I7—O13—Ba5	100.11 (15)
O14—Ba4—O15^xii^	94.97 (8)	Ba1^xiv^—O14—Ba1^vii^	88.31 (7)
O14—Ba4—O15^xiii^	94.97 (8)	Ba1^xiv^—O14—Ba1^viii^	88.31 (7)
O15^xiii^—Ba4—I7^viii^	31.07 (7)	Ba1^viii^—O14—Ba1^vii^	172.17 (14)
O15^xii^—Ba4—I7^vii^	31.07 (7)	Ba4—O14—Ba1^vii^	86.79 (7)
O15^xii^—Ba4—I7^viii^	139.91 (7)	Ba4—O14—Ba1^xiv^	99.25 (12)
O15^xiii^—Ba4—I7^vii^	139.91 (7)	Ba4—O14—Ba1^viii^	86.79 (7)
O15^xiii^—Ba4—O10^viii^	52.72 (9)	I6^xx^—O14—Ba1^viii^	93.81 (7)
O15^xii^—Ba4—O10^viii^	115.70 (9)	I6^xx^—O14—Ba1^vii^	93.81 (7)
O15^xiii^—Ba4—O10^ix^	115.70 (9)	I6^xx^—O14—Ba1^xiv^	102.16 (15)
O15^xii^—Ba4—O10^ix^	52.72 (9)	I6^xx^—O14—Ba4	158.60 (19)
O15^xiii^—Ba4—O15^xii^	168.25 (15)	Ba1^xiv^—O15—Ba4^xiii^	84.74 (7)
O8^ii^—Ba5—I6^ii^	88.99 (5)	Ba1^xiv^—O15—Ba4^xii^	84.74 (7)
O8^xiv^—Ba5—I6^ii^	31.42 (5)	Ba3^xx^—O15—Ba1^xiv^	128.21 (15)
O8^xv^—Ba5—I6^ii^	94.53 (5)	Ba3^xx^—O15—Ba4^xii^	91.21 (8)
O8^iii^—Ba5—I6^ii^	133.80 (5)	Ba3^xx^—O15—Ba4^xiii^	91.21 (8)
O8^xiv^—Ba5—O8^ii^	80.50 (8)	Ba4^xiii^—O15—Ba4^xii^	168.26 (15)
O8^xiv^—Ba5—O8^iii^	109.06 (5)	I7^xiv^—O15—Ba1^xiv^	108.21 (17)
O8^xv^—Ba5—O8^iii^	80.50 (8)	I7^xiv^—O15—Ba3^xx^	123.57 (18)
O8^xv^—Ba5—O8^ii^	109.06 (5)	I7^xiv^—O15—Ba4^xii^	94.12 (7)
O8^iii^—Ba5—O8^ii^	51.26 (10)	I7^xiv^—O15—Ba4^xiii^	94.12 (7)
O8^xiv^—Ba5—O8^xv^	69.24 (11)		
			
Ba1^xviii^—I6—O12—Ba2	93.51 (9)	Ba5—I7—O9—Ba1	−176.105 (13)
Ba1^xix^—I6—O12—Ba2	180.000 (1)	Ba5—I7—O9—Ba2	109.21 (8)
Ba1^xvi^—I6—O12—Ba2	−93.51 (9)	Ba5—I7—O9—Ba3^vi^	−5.76 (12)
Ba1^xvi^—I6—O12—Ba3	86.49 (8)	Ba5—I7—O9—Ba4^vi^	−97.19 (8)
Ba1^xviii^—I6—O12—Ba3	−86.49 (8)	Ba5—I7—O10—Ba3^vi^	88.20 (8)
Ba1^xix^—I6—O12—Ba3	0.000 (1)	Ba5—I7—O10—Ba4^vi^	172.43 (10)
Ba1^xix^—I6—O12—Ba5^vii^	−93.99 (8)	Ba5—I7—O13—Ba3^ii^	91.44 (8)
Ba1^xviii^—I6—O12—Ba5^vii^	179.517 (15)	Ba5—I7—O13—Ba3^vi^	−91.44 (8)
Ba1^xvi^—I6—O12—Ba5^vii^	−7.49 (16)	Ba5—I7—O13—Ba4	180.000 (1)
Ba1^xvi^—I6—O12—Ba5^viii^	−179.517 (15)	O8^xvi^—I6—O12—Ba2	−45.51 (8)
Ba1^xix^—I6—O12—Ba5^viii^	93.99 (8)	O8^xvii^—I6—O12—Ba2	45.51 (8)
Ba1^xviii^—I6—O12—Ba5^viii^	7.49 (16)	O8^xvi^—I6—O12—Ba3	134.49 (8)
Ba2—I7—O9—Ba1	74.68 (8)	O8^xvii^—I6—O12—Ba3	−134.49 (8)
Ba2—I7—O9—Ba3^vi^	−114.97 (12)	O8^xvi^—I6—O12—Ba5^viii^	−131.52 (11)
Ba2—I7—O9—Ba4^vi^	153.60 (14)	O8^xvii^—I6—O12—Ba5^viii^	−40.50 (11)
Ba2—I7—O10—Ba3^vi^	−21.57 (14)	O8^xvi^—I6—O12—Ba5^vii^	40.50 (11)
Ba2—I7—O10—Ba4^vi^	62.67 (11)	O8^xvii^—I6—O12—Ba5^vii^	131.52 (11)
Ba2—I7—O10—Ba5	−109.77 (8)	O9^i^—I7—O9—Ba1	52.70 (12)
Ba2—I7—O13—Ba3^ii^	−88.56 (8)	O9^i^—I7—O9—Ba2	−21.98 (16)
Ba2—I7—O13—Ba3^vi^	88.56 (8)	O9^i^—I7—O9—Ba3^vi^	−136.96 (6)
Ba2—I7—O13—Ba4	0.0	O9^i^—I7—O9—Ba4^vi^	131.61 (6)
Ba2—I7—O13—Ba5	180.0	O9—I7—O10—Ba3^vi^	−42.73 (10)
Ba3—I6—O12—Ba2	180.000 (2)	O9—I7—O10—Ba4^vi^	41.50 (10)
Ba3—I6—O12—Ba5^vii^	−93.99 (8)	O9—I7—O10—Ba5	−130.93 (10)
Ba3—I6—O12—Ba5^viii^	93.99 (8)	O9—I7—O13—Ba3^vi^	43.31 (12)
Ba3^vi^—I7—O9—Ba1	−170.35 (11)	O9^i^—I7—O13—Ba3^ii^	−43.31 (12)
Ba3^ii^—I7—O9—Ba1	94.88 (8)	O9^i^—I7—O13—Ba3^vi^	133.82 (11)
Ba3^ii^—I7—O9—Ba2	20.20 (14)	O9—I7—O13—Ba3^ii^	−133.82 (11)
Ba3^vi^—I7—O9—Ba2	114.97 (12)	O9^i^—I7—O13—Ba4	45.25 (8)
Ba3^ii^—I7—O9—Ba3^vi^	−94.78 (8)	O9—I7—O13—Ba4	−45.25 (8)
Ba3^ii^—I7—O9—Ba4^vi^	173.793 (17)	O9—I7—O13—Ba5	134.75 (8)
Ba3^vi^—I7—O9—Ba4^vi^	−91.43 (9)	O9^i^—I7—O13—Ba5	−134.75 (8)
Ba3^ii^—I7—O10—Ba3^vi^	94.58 (9)	O10—I7—O9—Ba1	−126.44 (10)
Ba3^ii^—I7—O10—Ba4^vi^	178.820 (19)	O10—I7—O9—Ba2	158.88 (12)
Ba3^vi^—I7—O10—Ba4^vi^	84.24 (8)	O10—I7—O9—Ba3^vi^	43.90 (11)
Ba3^ii^—I7—O10—Ba5	6.39 (12)	O10—I7—O9—Ba4^vi^	−47.53 (11)
Ba3^vi^—I7—O10—Ba5	−88.20 (8)	O10^i^—I7—O10—Ba3^vi^	138.13 (6)
Ba3^vi^—I7—O13—Ba3^ii^	−177.13 (16)	O10^i^—I7—O10—Ba4^vi^	−137.64 (6)
Ba3^ii^—I7—O13—Ba3^vi^	177.13 (16)	O10^i^—I7—O10—Ba5	49.93 (13)
Ba3^ii^—I7—O13—Ba4	88.56 (8)	O10^i^—I7—O13—Ba3^vi^	−136.90 (12)
Ba3^vi^—I7—O13—Ba4	−88.56 (8)	O10^i^—I7—O13—Ba3^ii^	45.97 (12)
Ba3^vi^—I7—O13—Ba5	91.44 (8)	O10—I7—O13—Ba3^ii^	136.90 (12)
Ba3^ii^—I7—O13—Ba5	−91.44 (8)	O10—I7—O13—Ba3^vi^	−45.97 (12)
Ba4^ii^—I7—O9—Ba1	10.90 (10)	O10—I7—O13—Ba4	−134.54 (8)
Ba4^vi^—I7—O9—Ba1	−78.92 (8)	O10^i^—I7—O13—Ba4	134.54 (8)
Ba4^vi^—I7—O9—Ba2	−153.60 (14)	O10^i^—I7—O13—Ba5	−45.46 (8)
Ba4^ii^—I7—O9—Ba2	−63.78 (11)	O10—I7—O13—Ba5	45.46 (8)
Ba4^ii^—I7—O9—Ba3^vi^	−178.751 (17)	O11^vii^—I6—O12—Ba2	−134.11 (8)
Ba4^vi^—I7—O9—Ba3^vi^	91.43 (9)	O11^x^—I6—O12—Ba2	134.11 (8)
Ba4^ii^—I7—O9—Ba4^vi^	89.82 (9)	O11^vii^—I6—O12—Ba3	45.89 (8)
Ba4^ii^—I7—O10—Ba3^vi^	−173.745 (18)	O11^x^—I6—O12—Ba3	−45.89 (8)
Ba4^vi^—I7—O10—Ba3^vi^	−84.24 (8)	O11^vii^—I6—O12—Ba5^viii^	139.88 (12)
Ba4^ii^—I7—O10—Ba4^vi^	−89.51 (9)	O11^vii^—I6—O12—Ba5^vii^	−48.10 (11)
Ba4^vi^—I7—O10—Ba5	−172.43 (10)	O11^x^—I6—O12—Ba5^viii^	48.10 (11)
Ba4^ii^—I7—O10—Ba5	98.06 (8)	O11^x^—I6—O12—Ba5^vii^	−139.88 (12)
Ba4^vi^—I7—O13—Ba3^vi^	4.44 (17)	O13—I7—O9—Ba1	143.33 (10)
Ba4^vi^—I7—O13—Ba3^ii^	−172.690 (16)	O13—I7—O9—Ba2	68.65 (12)
Ba4^ii^—I7—O13—Ba3^ii^	−4.44 (17)	O13—I7—O9—Ba3^vi^	−46.32 (11)
Ba4^ii^—I7—O13—Ba3^vi^	172.690 (16)	O13—I7—O9—Ba4^vi^	−137.75 (11)
Ba4^vi^—I7—O13—Ba4	−84.13 (9)	O13—I7—O10—Ba3^vi^	47.89 (11)
Ba4^ii^—I7—O13—Ba4	84.13 (9)	O13—I7—O10—Ba4^vi^	132.13 (10)
Ba4^ii^—I7—O13—Ba5	−95.87 (9)	O13—I7—O10—Ba5	−40.31 (10)
Ba4^vi^—I7—O13—Ba5	95.87 (9)	O15^iv^—I7—O9—Ba1	−34.96 (10)
Ba5^vii^—I6—O12—Ba2	−86.01 (8)	O15^iv^—I7—O9—Ba2	−109.64 (12)
Ba5^viii^—I6—O12—Ba2	86.01 (8)	O15^iv^—I7—O9—Ba3^vi^	135.39 (11)
Ba5^viii^—I6—O12—Ba3	−93.99 (8)	O15^iv^—I7—O9—Ba4^vi^	43.96 (11)
Ba5^vii^—I6—O12—Ba3	93.99 (8)	O15^iv^—I7—O10—Ba3^vi^	−130.39 (10)
Ba5^viii^—I6—O12—Ba5^vii^	172.02 (16)	O15^iv^—I7—O10—Ba4^vi^	−46.16 (10)
Ba5^vii^—I6—O12—Ba5^viii^	−172.02 (16)	O15^iv^—I7—O10—Ba5	141.41 (10)

Symmetry codes: (i) *x*, −*y*+1/2, *z*; (ii) −*x*+1/2, −*y*+1, *z*−1/2; (iii) −*x*+1/2, *y*−1/2, *z*−1/2; (iv) *x*−1/2, *y*, −*z*+1/2; (v) *x*−1/2, −*y*+1/2, −*z*+1/2; (vi) −*x*+1/2, −*y*, *z*−1/2; (vii) −*x*+1/2, −*y*+1, *z*+1/2; (viii) −*x*+1/2, −*y*, *z*+1/2; (ix) −*x*+1/2, *y*+1/2, *z*+1/2; (x) −*x*+1/2, *y*−1/2, *z*+1/2; (xi) *x*−1/2, *y*, −*z*+3/2; (xii) −*x*+1, −*y*+1, −*z*+1; (xiii) −*x*+1, −*y*, −*z*+1; (xiv) *x*+1/2, *y*, −*z*+1/2; (xv) *x*+1/2, −*y*+1/2, −*z*+1/2; (xvi) −*x*, −*y*+1, −*z*+1; (xvii) −*x*, *y*−1/2, −*z*+1; (xviii) −*x*, −*y*, −*z*+1; (xix) *x*, *y*, *z*+1; (xx) *x*+1/2, *y*, −*z*+3/2.
